# Hypomethylation-mediated upregulation of the *WASF2* promoter region correlates with poor clinical outcomes in hepatocellular carcinoma

**DOI:** 10.1186/s13046-022-02365-7

**Published:** 2022-04-28

**Authors:** Hye Ri Ahn, Geum Ok Baek, Moon Gyeong Yoon, Ju A Son, Jung Hwan Yoon, Jae Youn Cheong, Hyo Jung Cho, Ho Chul Kang, Jung Woo Eun, Soon Sun Kim

**Affiliations:** 1 Department of Gastroenterology, Ajou University School of Medicine, Worldcup-ro 164, Yeongtong-Gu, Suwon, 16499 Republic of Korea; 2 Department of Biomedical Sciences, Ajou University Graduate School of Medicine, Worldcup-ro 164, Yeongtong-Gu, Suwon, 16499 Republic of Korea; 3 Department of Pathology, College of Medicine, The Catholic University of Korea, 222 Banpo-daero, Seocho-gu, Seoul, 06591 Republic of Korea; 4 Department of Physiology, Ajou University Graduate School of Medicine, Worldcup-ro 164, Yeongtong-Gu, Suwon, 16499 Republic of Korea

**Keywords:** Carcinogenesis, Liver neoplasms, DNA methylation, Prognosis, WASF2

## Abstract

**Background:**

Hepatocellular carcinoma (HCC) is one of the most common and lethal cancers worldwide. Wiskott–Aldrich syndrome protein family member 2 (WASF2) is an integral member of the actin cytoskeleton pathway, which plays a crucial role in cell motility. In this study, we aimed to explore the role of *WASF2* in HCC carcinogenesis and its regulatory mechanism.

**Methods:**

*WASF2* expression in HCC was analyzed using six public RNA-seq datasets and 66 paired tissues from patients with HCC. The role of *WASF2* in normal hepatocyte cell phenotypes was evaluated using a *WASF2* overexpression vector in vitro; it was evaluated in HCC cell phenotypes using small interfering RNA (siRNA) in vitro and in vivo. Epigenetic regulatory mechanism of *WASF2* was assessed in the Cancer Genome Atlas liver hepatocellular carcinoma project (TCGA_LIHC) dataset and also validated in 38 paired HCC tissues. Site mutagenesis, bisulfite sequencing polymerase chain reaction (BSP), methylation-specific polymerase chain reaction (MSP), and quantitative MSP (qMSP) were used for evaluating *WASF2* methylation status.

**Results:**

*WASF2* is overexpressed in HCC and is clinically correlated with its prognosis. *WASF2* overexpression promoted normal hepatocyte proliferation. *WASF2* inactivation decreased the viability, growth, proliferation, migration, and invasion of Huh-7 and SNU475 HCC cells by inducing G2/M phase arrest. This induced cell death and inhibited epithelial–mesenchymal transition, hindering actin polymerization. In addition, *WASF2* knockdown using siWASF2 in a xenograft mouse model and a lung metastasis model exerted tumor suppressive effect. There was a negative correlation between *WASF2* methylation status and mRNA expression. The methylation pattern of CpG site 2 (− 726 bp), located in the *WASF2* promoter, plays an important role in the regulation of WASF2 expression. Furthermore, the cg242579 CpG island in the *WASF2* 5′ promoter region was hypomethylated in HCC compared to that in the matched non-tumor samples. Patients with high *WASF2* methylation and low *WASF2* expression displayed the highest overall survival.

**Conclusions:**

*WASF2* is overexpressed and hypomethylated in HCC and correlates with patient prognosis. *WASF2* inactivation exerts anti-tumorigenic effects on HCC cells in vitro and in vivo, suggesting that *WASF2* could be a potential therapeutic target for HCC.

**Supplementary Information:**

The online version contains supplementary material available at 10.1186/s13046-022-02365-7.

## Background

Hepatocellular carcinoma (HCC) is the most prevalent form of primary liver cancer, accounting for 70–85% of all cases [1]. Despite the recent development of targeted therapies and immunotherapies, HCC-related mortality has increased over the last three decades [2]. Moreover, there is a lack of standard molecular biomarkers for predicting treatment responses due to the phenotypic and molecular complexity and heterogeneity of HCCs [3]; therefore, it is important to understand the pathogenic mechanisms underlying HCC development and progression.

Wiskott–Aldrich syndrome protein family member 2 (WASF2), also known as WASP family verprolin-homologous protein 2, is an integral member of the actin cytoskeleton pathway and is activated by Ras-related C3 botulinum toxin substrate 1 (Rac1). The WASF2-actin-related protein 2/3 (Arp2/3) complex causes nucleation during actin assembly, leading to the formation of lamellipodia [4, 5]. WASF2 is involved in the invasion and metastasis of malignant B16F10 mouse melanoma cells [6] and the motility and invasion of pancreatic cancer cells (S2-013 and PANC-1) [7]. WASF2 is overexpressed in human HCC tissues compared to its levels in the surrounding non-tumor tissue and is positively correlated with poor overall survival (OS) [8]; however, the role of WASF2 in HCC cell phenotypes remains unclear. In addition, there is a lack of studies on the regulatory mechanism of WASF2, with the exception of one study in human umbilical vein endothelial cells and human aortic endothelial cells; it suggested microRNA-1253 as a novel regulator of WASF2 [9].

In this study, we confirmed that WASF2 overexpression in HCC tumor tissues is associated with poor clinical outcomes in patients with HCC. WASF2 suppression interrupts HCC cell growth, proliferation, migration, and invasion in vitro and in vivo by regulating the cell cycle, apoptosis, and epithelial–mesenchymal transition (EMT). CpG site 2 (− 726 bp) of cg24162579 CpG island methylation in the *WASF2* promoter could be an important mechanism for controlling WASF2 expression. Patients with cg24162579 hypomethylation display more aggressive tumor behavior and have a poorer prognosis than those with cg24162579 hypermethylation.

## Methods

### Patients and samples

Sixty-six pairs of frozen HCC tissues and corresponding non-cancerous liver tissues were obtained from the Ajou University Hospital (Suwon, Republic of Korea). Patient demographics and clinical characteristics are presented in Additional file 1: Table S1. All experiments were performed according to the Declaration of Helsinki and the study was approved by the Institutional Review Board of Ajou University Hospital (AJIRB-BMR-KSP-16-365 and AJIRB-BMR-SMP-17-189). Anonymous serum samples and clinical data were provided by the Ajou Human Bio-Resource Bank, and the need for informed consent was waived.

### 22 K human protein microarray

Protein microarray experiments were performed using a HuProt human proteome microarray v3.0 (CDI Laboratories Inc., Mayaguez, PR, USA). We obtained plasma samples from 15 human subjects: five healthy normal controls, five patients with liver cirrhosis, and five patients with HCC. From the patients with HCC, one sample per patient was obtained at 1 year before diagnosis, one at 6 months before diagnosis, and a third at diagnosis for a total of 15 samples. One sample was obtained from each healthy normal control and each patient with liver cirrhosis (10 samples in total). Taken together, we obtained 25 plasma samples. Briefly, each protein chip was equilibrated using microarray buffer (137 mM NaCl; 2.7 mM KCl; 4.3 mM Na_2_HPO_4_; 1.8 mM KH_2_PO_4_ pH 7.4; 0.05% Triton X-100) for 5 min at 22–25 °C and then incubated with 5% skim milk (BD Biosciences, San Jose, CA, USA) in microarray buffer for 1 h at 22–25 °C. To identify autoantibodies in HCC samples, a blocked protein chip was washed three times with microarray buffer for 10 min, incubated with 20 μg/mL of serum in reaction buffer (50 mM Tris-Cl pH 7.5; 2 mM DTT; 2.5 mM MgCl_2_) for 8 h at 4 °C, and then washed with microarray buffer for 10 min. The washed chip was then incubated with Alexa Fluor goat-anti rabbit 647-conjugated secondary antibodies (1:5000) diluted in microarray buffer containing 1% skim milk, for 30 min at 22–25 °C. The chip was then washed three times with microarray buffer, dried using centrifugation in a 50-mL conical tube (200×*g* for 2 min), and scanned using an Axon GenePix 4000B microarray scanner (Molecular Devices, San Jose, CA, USA). All spotted proteins were probed using glutathione S-transferase antibodies and Alexa Fluor goat-anti rabbit 546-conjugated secondary antibodies. The signal intensity of each spot was recorded as the ratio of foreground to background signal and normalized to that of glutathione S-transferase. The mean signal intensity of all proteins on the chip was calculated.

### Acquisition and analysis of the expression and methylation data in public omics databases

To analyze *WASF2* mRNA expression in HCC, we obtained expression datasets from The Cancer Genome Atlas liver hepatocellular carcinoma project (TCGA_LIHC), the International Cancer Genome Consortium liver cancer RIKEN Japan (ICGC_LIRI), and gene expression omnibus databases from the National Center for Biotechnology Information [GSE6764, GSE12443, GSE77314, GSE89377 (Catholic University of Korea’s liver hepatocellular carcinoma project, Catholic_LIHC), GSE93392, and GSE102418].

To analyze WASF2 protein expression in HCC, we obtained representative immunohistochemistry (IHC) images of the clinical specimens of patients with HCC from the Human Protein Atlas database (www.proteinatlas.org). According to the IHC intensity, samples were divided into positive and negative.

To analyze *WASF2* methylation status, we detected genome-wide DNA methylation in TCGA_LIHC. Level 3 data sets obtained using an Infinium HumanMethylation450 BeadChip were analyzed using R software (http://www.R-project.org/). The correlation between *WASF2* methylation and expression was analyzed and visualized using MEXPRESS [10].

### Ingenuity Pathway Analysis (IPA)

Signaling pathways downstream of WASF2 and its target genes were subjected to functional annotation of the actin cytoskeleton via IPA (Qiagen Inc., Redwood City, CA, USA).

### Cell culture, treatments, and transfection

Human HCC cell lines (Huh-7, Hep3B, PLC/PRF/5, SNU368, SNU398, SNU423, SNU449, and SNU475) were acquired from the Korean Cell Line Bank (Seoul, South Korea). Immortalized normal hepatocytes (MIHA) were provided by Dr. Roy-Chowdhury (Albert Einstein College of Medicine, Bronx, NY, USA). HCC and MIHA cells were cultured in RPMI 1640 or Dulbecco’s modified Eagle’s medium containing 10% fetal bovine serum (Invitrogen, Waltham, MA, USA) and 100 units/mL penicillin-streptomycin (GenDEPOT, Barker, TX, USA). THLE-2 immortalized normal hepatocytes were obtained from the American Type Culture Collection (ATCC, Bethesda, MD, USA) and cultured in bronchial epithelial cell growth medium (Lonza, Walkersville, MD, USA) supplemented with 10% fetal bovine serum (Invitrogen), 5 ng/mL epidermal growth factor (Sigma-Aldrich, St. Louis, MO, USA), 70 ng/mL phosphoethanolamine (Sigma-Aldrich), and antibiotics. Cells were grown in a humidified incubator with 5% CO_2_ at 37 °C.

Cells were starved in serum-free medium for 4 h prior to treatment with human recombinant transforming growth factor-β1 (TGF- β1; 20 ng/mL; R&D Systems, Minneapolis, MN, USA) dissolved in sterile 4 mM HCl supplemented with 0.1% bovine serum albumin (BSA; R&D Systems) for 24 h. The small interfering RNAs (siRNA) and negative control RNA duplexes were purchased from Bioneer (Daejeon, South Korea) and Genolution (Seoul, South Korea), respectively. pcDNA3.1-WASF2 and empty pcDNA3.1 vector were obtained from GenScript Biotech (Piscataway, NJ, USA). For transfection, cells were seeded into 60 mm dishes (2 × 10^5^ cells). When they reached 30 − 40% confluence, cells were transfected with 100 nM siRNAs or 1–2 μg plasmids using Lipofectamine 2000 (Invitrogen), according to the manufacturer’s instructions. Two days later, transfection efficiency was evaluated using quantitative real-time polymerase chain reaction (qRT-PCR) or western blot analysis.

### RNA isolation and quantitative real-time polymerase chain reaction analysis

Total RNA was isolated from frozen tissues and cell lines using QIAzol reagent (Qiagen, Hilden, Germany), according to the manufacturer’s instructions. cDNA was synthesized from 500 ng of total RNA in a 10 μL final volume using 5X PrimeScript™ RT Master Mix (Takara Bio, Shiga, Japan). qRT-PCR was performed using amfiSure qGreen Q-PCR Master Mix (GenDEPOT) and monitored in real time using a CFX Connect Real-Time PCR Detection System (Bio-Rad Laboratories, Hercules, CA, USA). The PCR conditions were as follows: 95 °C for 2 min, 40 cycles of 95 °C for 15 s, 58 − 62 °C for 34 s, and 72 °C for 30 s, followed by a dissociation stage at 95 °C for 10 s, 65 °C for 5 s, and 95 °C for 5 s. The sequences of the primers used are listed in Additional file 1: Table S2. All assays were performed three times.

### Western blot analysis

Proteins were extracted from tissues and cell lysates using radio immunoprecipitation buffer containing Halt™ Protease Inhibitor Cocktail (Thermo Fisher Scientific, Waltham, MA, USA). Protein concentration was determined using the bicinchoninic acid assay (Thermo Fisher Scientific). Equal amounts of total proteins were separated using sodium dodecyl sulfate polyacrylamide gel electrophoresis and transferred to polyvinylidene difluoride membranes (Merck Millipore, Burlington, MA, USA). The membranes were blocked with 5% skim milk in Tris-buffer saline and 0.1% Tween-20 for 1 h at 22–25 °C, incubated overnight with primary antibodies at 4 °C, and then incubated with HRP-conjugated secondary antibodies at 22–25 °C for 1 h. Chemiluminescence signals were detected using Clarity™ Western ECL Substrate (Bio-Rad Laboratories) and visualized using ChemiDoc™ (Bio-Rad Laboratories). The membrane band optical density was quantified using ImageJ software version 1.49 (Laboratory for Optical and Computational Instrumentation, Madison, WI, USA). The details of the antibodies are listed in Additional file 1: Table S3 and S4.

### Cell viability and growth assays

To analyze cell viability, MIHA and two HCC cell lines transfected for 72 h in 60 mm dishes were harvested through trypsinization, stained using 0.4% trypan blue solution (Invitrogen), and counted using a hemocytometer (Paul Marienfeld GmbH & Co. KG, Lauda-Königshofen, Germany).

To analyze cell growth, MIHA cells were seeded in 24-well plates, transfected with WASF2 overexpression vector (EX-WASF2) or negative control vector (Empty vector). HCC cells were seeded in 12-well plates, transfected with WASF2-targeting siRNA (siWASF2) or negative control siRNA (siNC). MIHA and HCC cells were incubated for 90 min at 37 °C with 0.5 mg/mL of 3-(4,5-dimethylthiazol-2-yl)-2,5-diphenyltetrazolium bromide (MTT; Biosesang, Seongnam, South Korea) solution. The dark blue formazan products were dissolved in dimethyl sulfoxide (Biosesang) and absorbance was detected using a TECAN SUNRISE Microplate Reader (TECAN, Zürich, Switzerland) at a wavelength of 570 nm.

### Clonogenic proliferation assay

MIHA cells were transfected with EX-WASF2 or Empty vector in 60 mm dishes for 48 h, reseeded in 6-well plates (6, 8, or 10 × 10^3^ cells per well), and incubated at 37 °C in a CO_2_ incubator for 10 days. HCC cells were transfected with siWASF2 or siNC in 60 mm dishes for 48 h, reseeded in 6-well plates (2, 4, or 6 × 10^3^ cells per well), and incubated at 37 °C in a CO_2_ incubator for 10 days. Cells were washed with phosphate-buffered saline (PBS), fixed with 1% paraformaldehyde for 30 min, stained with 0.5% crystal violet overnight at 22–25 °C, and counted using ImageJ software version 1.49 (Laboratory for Optical and Computational Instrumentation).

### Wound healing assay

MIHA cells were transfected with EX-WASF2 or Empty vector in 60 mm dishes for 48 h, reseeded in 6-well plates (1.5 × 10^6^ cells per well) and incubated at 37 °C in a CO_2_ incubator for 24 h. The cell monolayer was scratched manually using a sterile micropipette tip. Initial (0 h after scratching) and residual (24 h after scratching) gap widths were imaged using an Olympus CKX53 microscope (Olympus, Tokyo, Japan).

HCC cells were transfected with siWASF2 or siNC in 60 mm dishes for 48 h, reseeded in 6-well plates (1.5 × 10^6^ cells per well), and incubated at 37 °C in a CO_2_ incubator for 24 h. The cell monolayer was scratched manually using a sterile micropipette tip. Initial (0 h after scratching) and residual (48 h after scratching) gap widths were imaged using an Olympus CKX53 microscope (Olympus). Each area was measured thrice and the values were expressed as a percentage.

### Apoptosis and cell cycle assays

Apoptosis was detected using an Annexin V-FITC Apoptosis Detection Kit (Koma Biotech, Seoul, South Korea). Briefly, cells transfected for 48 h were harvested through trypsinization, rinsed in PBS, and resuspended in 1× binding buffer before being incubated with 1.25 μL of Annexin V-FITC solution at 22–25 °C for 20 min in the dark, stained with 10 μL propidium iodide (PI; Sigma-Aldrich), and analyzed using a FACSAria III flow cytometer (BD Biosciences).

Apoptotic cells were evaluated using Hoechst 33342 and PI staining. The transfected cells were fixed in 4% paraformaldehyde (Biosesang) for 10 min and permeabilized with 0.2% Triton X-100 (Sigma-Aldrich) in PBS for 20 min. The cells were then treated with Hoechst 33342 (10 μg/mL; Invitrogen) for 10 min, stained with PI (20 μg/mL; Sigma-Aldrich) for 20 min, and observed using an Olympus IX71 microscope (Olympus).

To analyze the cell cycle, transfected cells were collected, fixed using 70% ethanol for 3 h at 4 °C, washed in PBS, and stained using 200 μL PBS containing 10 μg/mL RNase A, 1% Triton X-100, and 30 μg/mL PI (Sigma-Aldrich) for 30 min in the dark. The proportion of cells at each stage of the cell cycle was determined using a FACSAria III flow cytometer (BD Biosciences). Experiments were performed in triplicate.

### Migration and invasion assays

For the in vitro cell migration and invasion assays, we used 24-well plates and cell culture inserts (BD Biosciences). Prior to the invasion assay, the upper compartment of the cell culture insert was coated with 100 μL of Matrigel (BD Biosciences) diluted to 0.3 mg/mL in serum-free media. Huh-7 and SNU475 cells were transfected with siWASF2 or siNC for 24 h; then, they were seeded onto the cell culture insert with serum-free media and 5–20% fetal bovine serum, as a chemoattractant, for 24–72 h at 37 °C in a 5% CO_2_ humidified incubator. Migratory or invasive cells on the lower side of the membrane were stained using a Diff-Quik staining kit (Sysmex Corporation, Chuo-ku, Japan) and imaged using a CKX53 inverted microscope (Olympus) at 200× magnification. Cells were counted in three random fields of view.

### Immunofluorescence

Cells were seeded in 24-well plates and fixed in 4% paraformaldehyde (Biosesang) for 10 min at 22–25 °C and permeabilized in PBS with 0.2% Triton X-100 (PBST; Sigma-Aldrich) for 10 min. The cells were washed in PBST; the plates were blocked with 2.5% BSA in PBST for 30 min and incubated with the primary antibody against F-actin (1:200; Abcam, Waltham, MA, USA). The plates were washed and incubated with Alexa Fluor-488-conjugated secondary antibodies for 2 h and stained with 4′,6-diamidino-2-phenylindole. The stained plates were analyzed using Olympus IX71 microscope (Olympus).

### Animal experiments

Five-week-old BALB/c female nude mice were purchased from ORIENT BIO (Seongnam, South Korea) and six-week-old athymic female nude mice were purchased from Koatech (Pyeongtaek, South Korea). They were placed in individually ventilated cages under a pathogen-free environment, and allowed to acclimate for 2 weeks before being used in experiments.

For the in vivo subcutaneous xenograft assay, Huh-7 cells transfected with siWASF2 or siNC (5 × 10^5^ cells) were diluted in 0.1 mL Matrigel/serum-free Dulbecco’s modified Eagle’s medium and injected subcutaneously into the flanks of BALB/c nude mice. Tumor diameter was measured three times a week for 18 days in the tumor-bearing mice using a digital caliper. Tumor volume was calculated as follows: *Tumor volume* = 0.52 × *length* × *width*^2^.

At the end of the experimental period, tumors were removed for weighing and histological analysis. Tumor tissue samples were immediately flash-frozen in liquid nitrogen for RNA and protein extraction and placed in 10% neutral buffered formalin for hematoxylin and eosin (H&E) staining and IHC.

For in vivo lung metastasis assay, *ras*-transformed NIH-3 T3 cells transfected with siWASF2 or siNC (3 × 10^5^ cells) were mixed with 0.2 mL serum-free Dulbecco’s modified Eagle’s medium and injected into the athymic nude mice through tail veins. The mice were sacrificed 14 days after cell inoculation, and the number of the peritoneal nodules were assessed.

All animals were maintained according to the Guide for the Care and Use of Laboratory Animals; the experiments were approved by the Ethics Committee for Laboratory Animal Research Center of Ajou University Medical Center (IACUC-2020-0007).

### IHC

Tissue samples from patients with HCC were fixed in formalin, embedded in paraffin, and cut into 5-μm sections that were deparaffinized in xylene, hydrated in graded alcohol, and incubated with primary antibodies (listed in Additional file 1: Table S3) overnight at 4 °C. After being washed three times, the sections were incubated with secondary antibodies for 1 h and then a peroxidase substrate until the desired stain intensity developed.

In the BALB/c nude mouse model, tumors were harvested, fixed in 10% neutral buffered formalin, and embedded in paraffin blocks before being cut into 5-μm sections and deparaffinized. One section was stained with H&E and another was evaluated using IHC. Both sections were incubated with the antibodies listed in Additional file 1: Table S3.

### Plasmid constructions

The *WASF2* promoter construct (spanning − 833/− 534 and containing four CpG sites) was generated through PCR amplification using genomic DNA from Hep3B cells, and cloned into pGL3-basic vector (E1751, Promega, Madison, WI, USA) using KpnI and XhoI. The following PCR primers specific for the *WASF2* promoter: 5′-GGTACCGTGAGCGGAACCCTGGTTCAGC-3′ (forward) and 5′-CTCGAGGGGGTTTCTCCAAGTTGGTCAGG-3′ (reverse) were used. The resultant PCR products were digested with the restriction enzymes and cloned into the multiple cloning site of a pGL3-basic vector using Rapid DNA Ligation Kit (Thermo Scientific).

### Mutagenesis

Point mutations at CpG site 1 or 2 in the *WASF2* promoter constructs were generated by converting CG to TG using the QuickChange II Site-Directed Mutagenesis Kit (Agilent Technologies, Santa Clara, CA, USA). The primers used for mutagenesis were designed using QuickChange Primer Design software (Agilent Technologies); the sequences of the primers were as follows: 5′- TGGGGAATATGCTTGTCTTGTTGA − 3′ (forward) and 5′- TTATAATCAACAAGACAAGCATATTC − 3′ (reverse) for Site 1, 5′-ATTATAAAAGGCTGCAGCTGTCTGGGCGCG-3′ (forward) and 5′- TACAGGCATGAGCCACCGCGCCCAGACAGC-3′ (reverse) for Site 2. *WASF2* promoter constructs with mutation (CG to TG) of CpG sites located at − 726 bp from the TSS were generated according to the manufacturer’s instructions. Construct sequences were confirmed through DNA sequencing using the ABI 3500 Genetic Analyzer (Applied Biosystems, Foster City, CA, USA).

### Bisulfite Sequencing Polymerase chain reaction (BSP)

Genomic DNA was isolated from human liver tissues and cell lines using a DNeasy Blood & Tissue kit (Qiagen), according to the manufacturer’s instructions. The DNA quality and concentration of each sample was checked using a NanoPhotometer® N60 (Implen, Munich, Germany) and 1 μg of DNA was modified using an EZ DNA Methylation-Gold kit (Zymo Research, Irvine, CA, USA), according to the manufacturer’s protocol. Briefly, DNA was heat-denatured and bisulfite-converted using CT-conversion reagent in a thermocycler. The DNA was bound to a Zymo-spin IC column and desulfonated using M-desulfonation buffer. After being eluted from the column using 10 μL of M-elution buffer, 3 μL of bisulfite-converted DNA was used for BSP to amplify the specific *WASF2* promoter region (cg24162579) using TaKaRa EpiTaq HS (Takara Bio) with a T100 Thermal Cycler (Bio-Rad Laboratories). The primers used to detect WASF2 CpG island methylation were designed using MethPrimer 2.0 (https://www.urogene.org/cgi-bin/methprimer/methprimer.cgi; Additional file 1: Table S2). The PCR cycling conditions were as follows: 40 cycles of 98 °C for 10 s, 63 °C for 60 s, and 72 °C for 30 s. The PCR products were cloned into a pTOP vector and transformed into DH5α-competent *E. coli* cells using a TOPcloner TA kit (Enzynomics, Daejeon, Korea). Five clones per sample were expanded overnight and plasmid DNA was extracted using a QIAGEN Plasmid Mini kit (Qiagen). Purified plasmid DNA was sequenced using an ABI 3730xl DNA analyzer (Applied Biosystems) with an M13 primer to analyze the methylation status of specific CpG sites.

### Methylation-specific polymerase chain reaction (MSP) and quantitative MSP (qMSP)

MSP and qMSP were performed using primers for methylated or unmethylated DNA designed using MethPrimer. Briefly, 2 μL of bisulfite-treated genomic DNA was amplified using TaKaRa EpiTaq HS (Takara Bio) under the following cycling conditions: 40 cycles of 98 °C for 10 s, 60 °C for 40 s, and 72 °C for 30 s. The PCR products were analyzed using 2% agarose gel electrophoresis. qMSP was performed using amfiSure qGreen Q-PCR Master Mix (GenDEPOT) and was monitored in real-time using an ABI 7300 Real-Time PCR System (Applied Biosystems). The cycling conditions were as follows: 95 °C for 2 min, followed by 40 cycles of 95 °C for 15 s, 60 °C for 34 s, and 72 °C for 30 s, followed by a single cycle of 95 °C for 15 s, 60 °C for 60 s, and 95 °C for 15 s to generate dissociation curves. Relative DNA methylation was calculated using the difference between the Ct values of the methylated and unmethylated PCR products. All measurements were performed in triplicate. The primer sequences used for MSP and qMSP are listed in Additional file 1: Table S2.

### Hydroxyurea (HU) and 5-aza-2′-deoxycytidine (5-aza) treatment

Cells were treated with 0.5 mM HU (Sigma-Aldrich) or 5 μM 5-aza (Sigma-Aldrich) for 48–72 h at 37 °C in a CO_2_ incubator, with the culture medium replaced daily. The treated cells were harvested and used to detect *WASF2* methylation and expression.

### Statistical analyses

Receiver operating characteristic (ROC) curves were produced using SPSS version 22 (SPSS, Chicago, IL, USA). OS and disease-free survival (DFS) curves were plotted using Graphpad™ 8.0 software (GraphPad Software, San Diego, CA, USA) using the Kaplan–Meier product limit method. Significant differences between survival curves were determined using the log-rank test. All experiments were performed at least thrice; all samples were analyzed in triplicate. Results are presented as mean ± standard error of the mean (SEM). Significant differences between experimental groups were assessed using paired or unpaired Welch’s *t*-tests and one-way analysis of variance (ANOVA) in Graphpad™ 8.0 software. *P* values < 0.05 were considered statistically significant.

## Results

### Identification of potential driver markers of HCC development using proteomic and transcriptomic databases

**Fig. 1:**
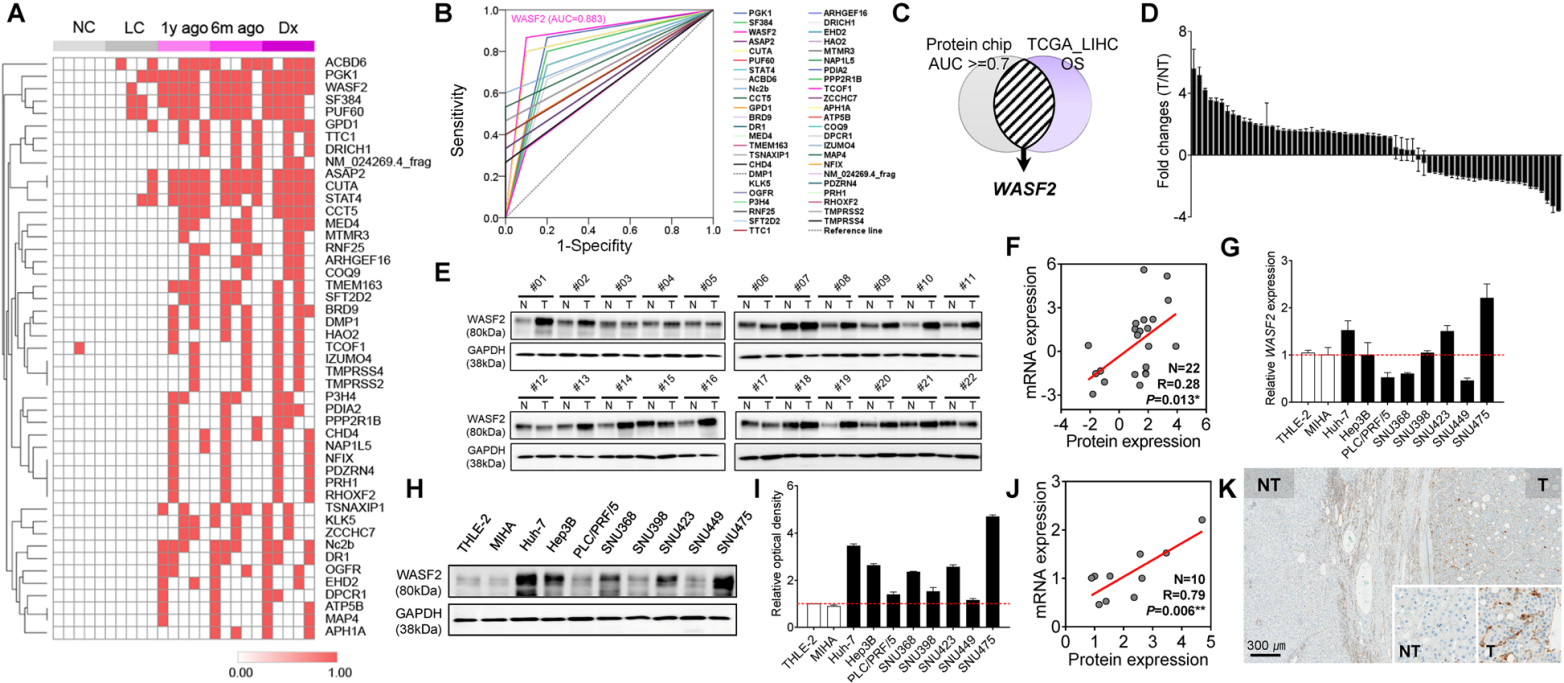
Identification of potential HCC driver markers using a 22 K human protein microarray system and clinical relevance of *WASF2* overexpression. **A** Heatmap of 47 differentially expressed autoantibodies from normal and liver cirrhosis to HCC (NC, normal control; LC, liver cirrhosis; 1 y ago, 1 year ago; 6 m ago, 6 months ago; Dx, HCC diagnosis). **B** ROC analysis of 47 autoantibodies. **C** Venn diagram analysis of the ROC result for 47 autoantibodies and OS from TCGA_LIHC. **D** Bar chart shows the fold change in tumor (T) versus non-tumor (NT) *WASF2* expression in 66 matched pairs of human HCC tissues and corresponding noncancerous adjacent liver tissues. **E** Western blot analysis of WASF2 in 22 matched pairs of human HCC tissues with corresponding noncancerous adjacent liver tissues. **F** Correlation analysis between mRNA and protein expression of WASF2 in HCC tissues (*n* = 22, Pearson’s correlation coefficient, *r* = 0.28, *P* = 0.013). **G** WASF2 mRNA and protein expression in two immortalized non-transformed hepatocyte cells (THLE-2 and MIHA) and eight HCC cell lines analyzed using qRT-PCR and **H** western blot analysis. **I** Graph chart shows the ratio of the relative density of WASF2 protein expression normalized to that GAPDH. **J** Correlation analysis between WASF2 mRNA and protein expression in normal liver cells and HCC cell lines (*n* = 10, Pearson’s correlation coefficient, *r* = 0.79, *P* = 0.006). **K** Representative IHC photomicrographs of WASF2 in HCC (T, tumor tissues; NT, non-tumor tissues). Scale bar = 300 μm. *P < 0.05; **P < 0.01

To identify potential early diagnostic markers in hepatocarcinogenesis, we analyzed protein and gene expression using the following systemic strategies. Plasma samples from five healthy normal controls, five patients with liver cirrhosis and five patients with early HCC (at diagnosis, 6 months before diagnosis, and 1 year before diagnosis) were analyzed using a 22 K protein chip. All subjects were male with a liver cirrhosis or HCC etiology involving hepatitis B virus. Serial clustering analysis revealed the positive expression of 47 autoantibodies from liver cirrhosis to HCC. Most autoantibodies were not detected in the normal control group (NC) and liver cirrhosis group (LC); however, all of them were differentially expressed in the 1 y ago group (1 year before HCC diagnosis), 6 m ago group (6 months before HCC diagnosis), and Dx group (at HCC diagnosis; Fig. 1A).

To select more precise diagnostic biomarkers for HCC, we performed ROC curve comparative analysis (Fig. 1B). Out of the 47 autoantibodies, 23 were significantly specific and sensitive as biomarkers, with an area under the curve (AUC) of > 0.7 (Additional file 1: Table S5). Venn diagram analysis performed with this ROC result and the OS analysis of TCGA_LIHC identified one putative candidate as early diagnostic marker for HCC: *WASF2* (Fig. 1C).

### WASF2 overexpression is closely related to the clinical outcome of HCC

First, we examined *WASF2* expression in large cohorts of HCC patients from TCGA_LIHC, ICGC_LIRI, and National Center for Biotechnology Information gene expression omnibus databases. Notably, *WASF2* was significantly overexpressed in HCC compared to that in non-tumor samples in three large cohort datasets (TCGA_LIHC, ICGC_LIRI, and GSE77314; Additional file 2: Fig. S1A). It was remarkably overexpressed in early HCC compared to its levels in non-cancerous samples in multistage datasets of hepatocarcinogenesis (GSE6764, GSE12443, and GSE114564; Additional file 2: Fig. S1B). We validated *WASF2* expression using qRT-PCR in a set of 66 pairs of HCC tissues, among which 36 (54.5%) showed *WASF2* overexpression (Fig. 1D). Increased WASF2 protein expression was evaluated using western blots in 22 randomly selected human HCC tissues in comparison to that in the corresponding non-cancerous liver tissues; WASF2 was overexpressed in 16 pairs (72.7%; Fig. 1E). The mRNA and protein expression levels of WASF2 for the same patient showed a high positive correlation (Pearson’s correlation coefficient *r* = 0.28, *P* = 0.013; Fig. 1F). Therefore, WASF2 overexpression in HCC was confirmed in the samples from both the public omics databases and our cohort. To select HCC cell lines overexpressing WASF2, we measured endogenous WASF2 levels using qRT-PCR and western blot in ten different liver cell lines, including in the immortalized normal hepatic cell lines, THLE-2 and MIHA. Five of the eight HCC cell lines exhibited high WASF2 expression compared to that in the normal hepatocytes; in particular, SNU475 and Huh-7 had the highest WASF2 expression and were selected for further experiments (Fig. 1G, H, I). The mRNA and protein expression levels of WASF2 for the same cell line showed a high positive correlation (Pearson’s correlation coefficient *r* = 0.79, *P* = 0.006; Fig. 1J) We performed IHC analysis on non-tumor and tumor tissues from patients, in cases where archived tissue was available, to confirm the expression of the putative marker, WASF2, indicated as brown spots corresponding to positive antibody staining. Subsequently, WASF2 was expressed positively in tumor tissue compared to that in non-tumor tissue (Fig. 1K). To further validate these results, the expression level of WASF2 protein in HCC tissues from Human Protein Atlas (HPA; https://www.proteinatlas.org/) was evaluated; 75% of the HCC tissues showed positive expression of WASF2 (Additional file 2: Fig. S1C, D). *WASF2* expression was upregulated in all HCC data sets; therefore, we assessed the association between *WASF2* expression and the prognosis of HCC patients in the TCGA_LIHC datasets. Kaplan–Meier survival analysis indicated that patients with high WASF2 expression displayed significantly lower 5-year OS, disease specific survival, and progression-free survival rates than those with low *WASF2* expression (Fig. S1E).

### Overexpression of WASF2 promotes hepatocyte proliferation and deactivation of WASF2 results in tumor suppression in vitro

**Fig. 2:**
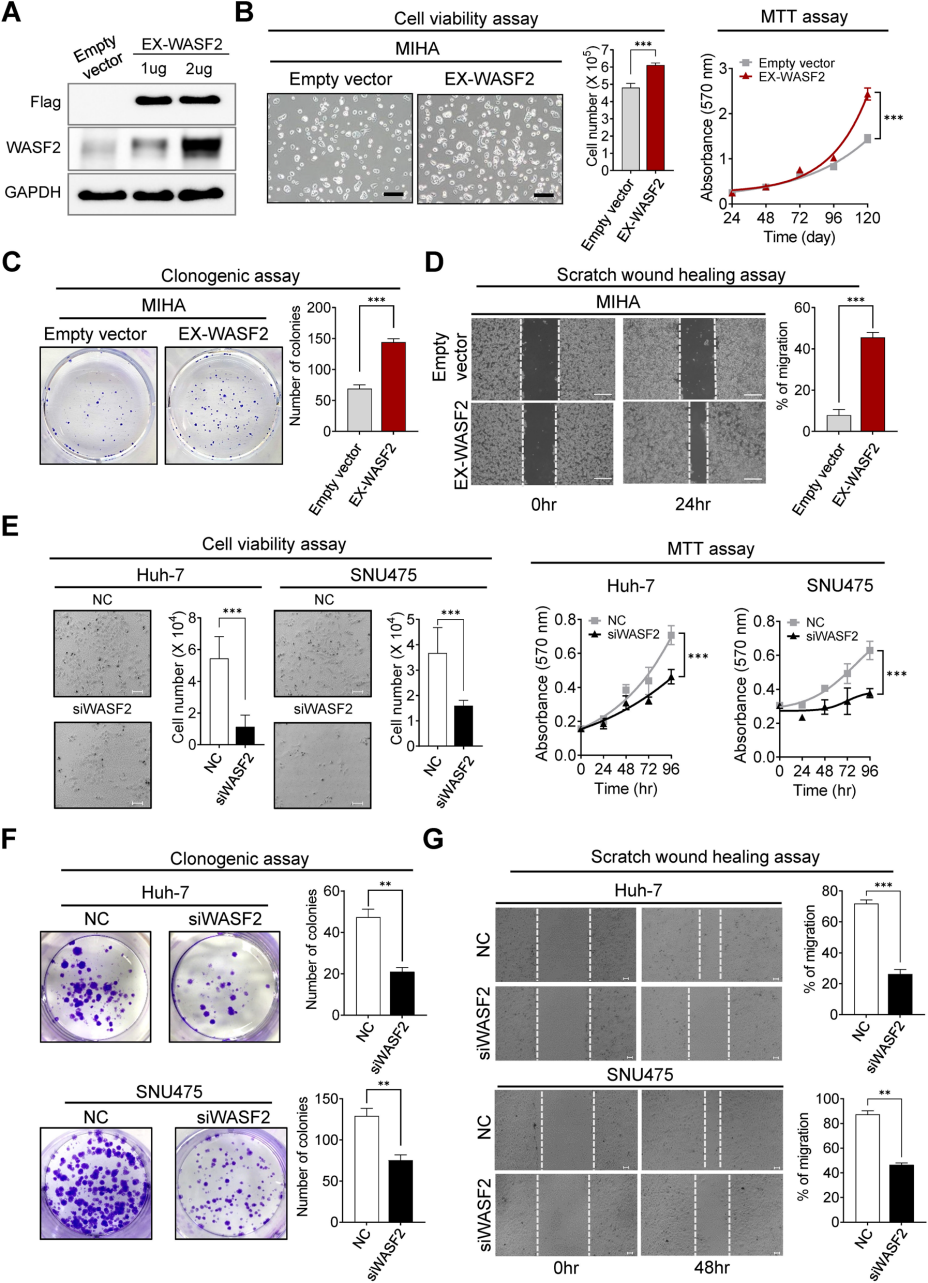
The oncogenic effect of *WASF2* overexpression in normal hepatocytes and the anti-tumorigenic effects of *WASF2* knockdown in HCC cells. **A** Representative western blot of the Flag and WASF2 expression in MIHA cells transfected with Empty vector or different concentrations of EX-WASF2. **B** Left: Representative morphology and number of MIHA cells transfected with Empty vector or EX-WASF2. Scale bar = 100 μm. Cell number was counted using trypan blue (mean ± SEM, *n* = 3, unpaired *t*-test). Right: Cell growth was measured using MTT assay. (mean ± SEM, *n* = 3, unpaired *t*-test). **C** Clonogenic and **D** scratch wound healing assays were performed in MIHA cells transfected with Empty vector or EX-WASF2. (mean ± SEM; *n* = 3, unpaired *t*-test). Scale bar = 100 μm. **E** Growth analyses. Left: morphology and number of Huh-7 and SNU475 cells transfected with NC or siWASF2. Scale bar = 100 μm. Cell number was counted using trypan blue (mean ± SEM, *n* = 3, unpaired *t*-test). Right: MTT assay (mean ± SEM, *n* = 3, unpaired *t*-test). **F** Clonogenic and **G** scratch wound healing assay in Huh-7 and SNU475 cells. Clonogenic assay: left, representative colony images; right, graphical representation of colony number from three random images (mean ± SEM; *n* = 3). Wound healing assay: left, representative images; right, graphical representation of the percentage of migrated cells from three random images (mean ± SEM; *n* = 3, unpaired *t*-test). Scale bar = 10 μm. **P < 0.01; ***P < 0.001

We investigated the potential oncogenic role of WASF2 in HCC. To investigate the effects of overexpression of WASF2 on normal hepatocytes, cell viability, MTT assay, clonogenic, and scratch wound healing assays were performed in MIHA cells overexpressing WASF2. First, we assessed the efficiency of the WASF2 overexpression vector (EX-WASF2) in MIHA cells (Fig. 2A). The growth rates of the MIHA cell transfected with empty vector and EX-WASF2 was assessed using cell viability and MTT assays. WASF2 overexpression significantly improved the viability and growth of MIHA cells (Fig. 2B). Clonogenic and scratch wound healing assays were performed to assess the rate of proliferation and the wound healing ability. WASF2 overexpression significantly induced the proliferation and serum-stimulated wound-healing efficacy of MIHA cells (Fig. 2C, D). To further understand the biological functions of WASF2 in HCC, its expression was reduced using RNA interference. We assessed the efficiency of three siRNAs targeting *WASF2* in Huh-7 and SNU475 cells, and selected the siRNA with the highest efficiency for further analyses (Additional file 2: Fig. S2A, B). The growth rates of the two HCC cell lines transfected with negative control siRNA and siWASF2 for 96 h were assessed using cell viability and MTT assays. *WASF2* knockdown significantly reduced the viability and growth of Huh-7 and SNU475 cells (Fig. 2E). Clonogenic and scratch wound healing assays indicated that *WASF2* knockdown significantly reduced the proliferation and serum-stimulated wound-healing efficacy of Huh-7 and SNU475 cells (Fig. 2F, G).

### Gene signatures correlated with WASF2 expression are associated with poor prognosis in patients with HCC

We confirmed WASF2 overexpression at both the mRNA and protein levels in HCC. We assessed the detailed prognostic relevance of *WASF2* expression in TCGA_LIHC dataset. Gene signatures with expression patterns that correlated highly with *WASF2* expression (*n* = 1354) were selected for cluster analysis (*P* < 0.001; *r* > 0.3 or *r* < 0.3). The HCC patients were divided into *WASF2* high- (*WASF2*_high) and *WASF2* low- (*WASF2*_low) expression groups (Fig. S3A). The heat map showed two distinct clusters; therefore, we performed survival analyses for the two groups. Kaplan–Meier survival analysis indicated that patients with HCC who displayed high *WASF2* expression had significantly lower 5-year OS and DFS rates than those with low *WASF2* expression (OS, log-rank *p* < 0.0001, hazard ratio [HR] = 2.31; DFS, log-rank *p* < 0.0001, HR = 1.97; Fig. S3B).

To elucidate the functional role of the gene signatures that correlated with *WASF2* expression, we performed gene set enrichment analysis (GSEA) using The Molecular Signatures Database (MSigDB). *WASF2*-related gene signatures were enriched in the majority of the cancer-associated pathways; G2M_CHECKPOINT was identified as a highly significant enriched gene set (Fig. S3C). The four representative enrichment plots revealed that these *WASF2*-related genes were significantly associated with the cell cycle, liver cancer survival, liver cancer proliferation, and HCC metastasis (Fig. S3D).

### Targeted WASF2 suppression elicits cell arrest, death, and reduced metastatic potential in HCC

**Fig. 3:**
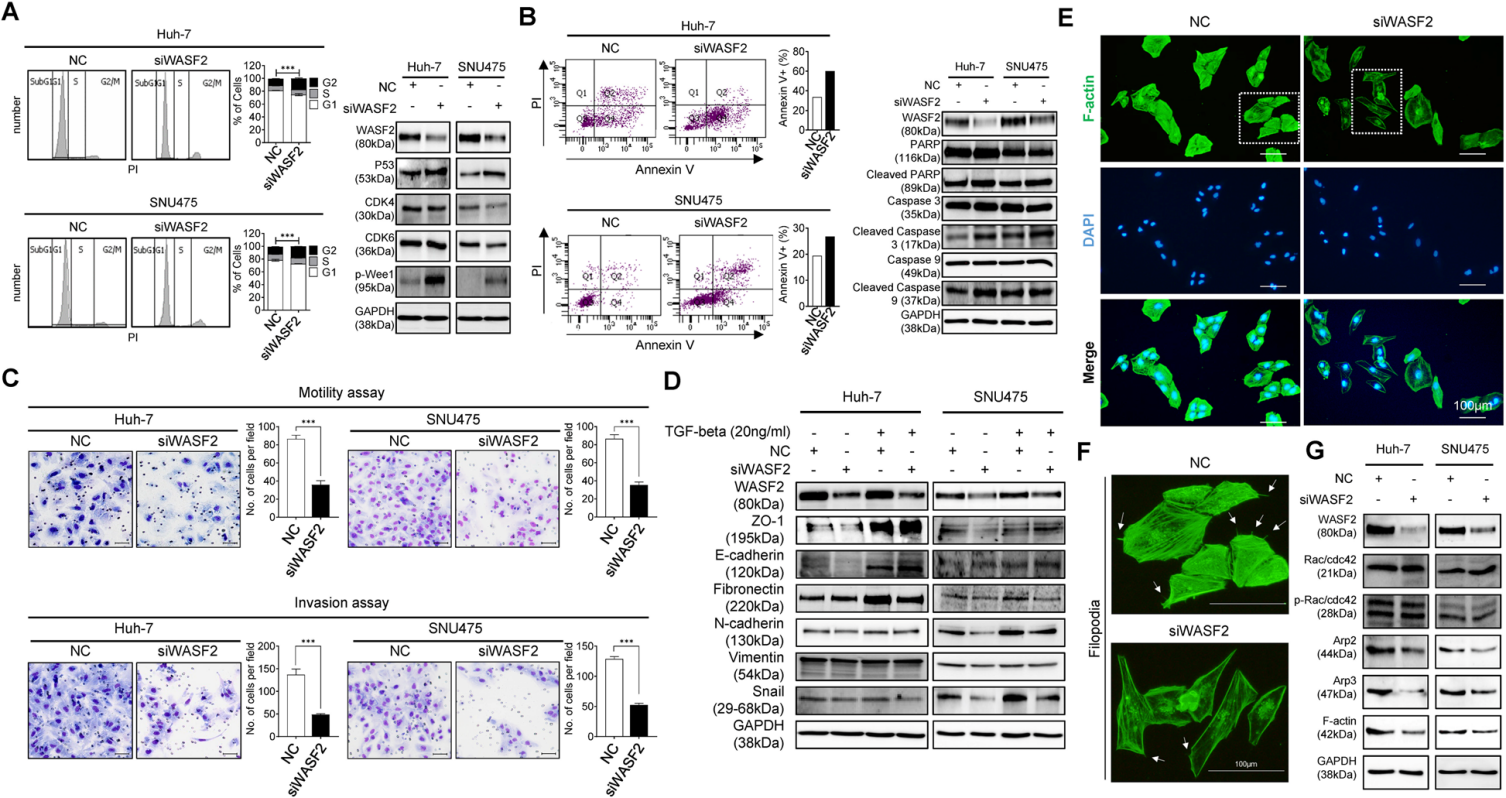
Regulatory effect of *WASF2* on cell cycle, apoptosis, EMT, and actin polymerization. **A** Cell cycle analysis in Huh-7 and SNU475 cells following siWASF2 transfection, using flow cytometry. The percentage of cells in G2/M phase was analyzed using a FACSAria III flow cytometer. Western blot analyses of cell-cycle modulators in two HCC cell lines (mean ± SEM; *n* = 3, unpaired *t*-test). **B** Apoptosis analysis using flow cytometry. Dot plots indicate apoptosis ratios using PI and FITC-annexin V (left). Bar graph shows average percentage of apoptotic cells (right). Western blot analysis of PARP, cleaved PARP, caspase-3, cleaved caspase-3, caspase-9, and cleaved caspase-9. GAPDH was used as the loading control. **C** Representative cell images and bar charts showing the number of migrating and invading cells transfected with siWASF2 captured in three random fields following modified Boyden chamber motility assay (top) and Transwell invasion assay (bottom; mean ± SEM; *n* = 3, unpaired *t*-test). Scale bar = 50 μm. **D** Western blot analysis of EMT molecules in the presence or absence of TGF-β1 (20 ng/mL) in Huh-7 and SNU475 cells. **E** IF staining of F-actin and **F** Filopodia formation (white arrows) in SNU475 cells transfected with negative-control siRNA (NC) or siWASF2. Representative fluorescence images are shown. Scale bar = 100 μm. **G** Western blot analysis of molecules related to the actin cytoskeleton in Huh-7 and SNU475 cells. ***P < 0.001

The G2M checkpoint was the most significant function associated with the *WASF2*-related gene signature in MSigDB; therefore, we performed flow cytometry cell-cycle analysis. *WASF2* knockdown significantly increased the proportion of cells in the G2/M phase and altered the expression of cell-cycle regulatory proteins including decreased cyclin-dependent kinase 4 and 6, and increased p53 and p-Wee1 (Fig. 3A). Therefore, we analyzed cell death using Annexin V-FITC/PI staining after *WASF2* knockdown, with subsequent FACS analysis. *WASF2* knockdown induced significant apoptosis in Huh-7 and SNU475 cells. In addition, *WASF2* knockdown upregulated the cell death regulators, such as cleaved poly ADP-ribose polymerase (PARP), cleaved caspase-3, and cleaved caspase-9 in HCC cells (Fig. 3B). Additionally, Hoechst 33342 and PI staining were used to assess morphological changes. *WASF2* knockdown increased the proportion of apoptotic and necrotic Huh-7 and SNU475 cells (Additional file 2: Fig. S4).

GSEA showed that the *WASF2*-related gene signatures were also enriched in the malignant features of HCC cells; therefore, we conducted in vitro migration and invasion assays to identify the role of WASF2 in the progression of HCC. Modified Boyden chamber assays revealed that *WASF2* knockdown significantly suppressed the migratory and invasive responses of both Huh-7 and SNU475 cells (Fig. 3C). To elucidate the regulatory effect of *WASF2* on EMT, we detected the expression of EMT regulatory proteins in HCC cells stimulated with TGF-β1 (20 ng/mL) using western blot analysis. *WASF2* knockdown increased the expression of zonula occuludens-1 (ZO-1) and E-cadherin in Huh-7 and SNU475 cells; however, it decreased the expression of fibronectin and Snail (Fig. 3D). A positive correlation was observed between the expression of *SNAI1* and *WASF2* in the tumor group of the TCGA_LIHC dataset (Pearson’s correlation coefficient *r* = 0.26, *P* < 0.0001), but not in the non-tumor group (Pearson’s correlation coefficient *r* = 0.08, *P* = 0.55; Fig. S5).

WASF2 is a key molecule modulating the actin cytoskeleton and reconstruction; it influences the migration and invasion of cancer cells [11]. To identify if WASF2 is associated with the actin signaling pathway, we analyzed the actin cytoskeleton signaling pathway related to WASF2 using IPA. WASF2 has a positive correlation with Arp2/3 and F-actin and subsequently causes actin polymerization (Additional file 2: Fig. S6). The cell motility characteristics and expression of F-actin were visualized through immunofluorescence staining. The levels of several proteins related to the actin cytoskeleton were evaluated through western blot analysis. In immunofluorescence staining, *WASF2* knockdown reduced the formation of filopodia around the cells and downregulated F-actin (Fig. 3E, F). Rac and cell division control protein 42 homolog (Cdc42) are Ras-related GTP-binding proteins that control the actin cytoskeleton; Arp2 and Arp3 are involved in the initiation of actin polymerization [12]. The actin network is a dynamic structure with continuous assembly and disassembly. F-actin, which plays an important role in actin polymerization, is a polymer of actin [13]. Arp2, Arp3, and F-actin levels were notably decreased in the *WASF2* knockdown group compared to those in the negative control group. The expression levels of Rac/Cdc42 was consistent after *WASF2* knockdown; in addition, the levels of the phosphorylated form (p-Rac/Cdc42) remained stable (Fig. 3G). Therefore, the dynamic reorganization of actin is a prerequisite for the migration of cancer cells; WASF2 is involved in the construction of the complex actin cytoskeleton.

### WASF2 suppression attenuates HCC tumorigenesis in vivo

**Fig. 4:**
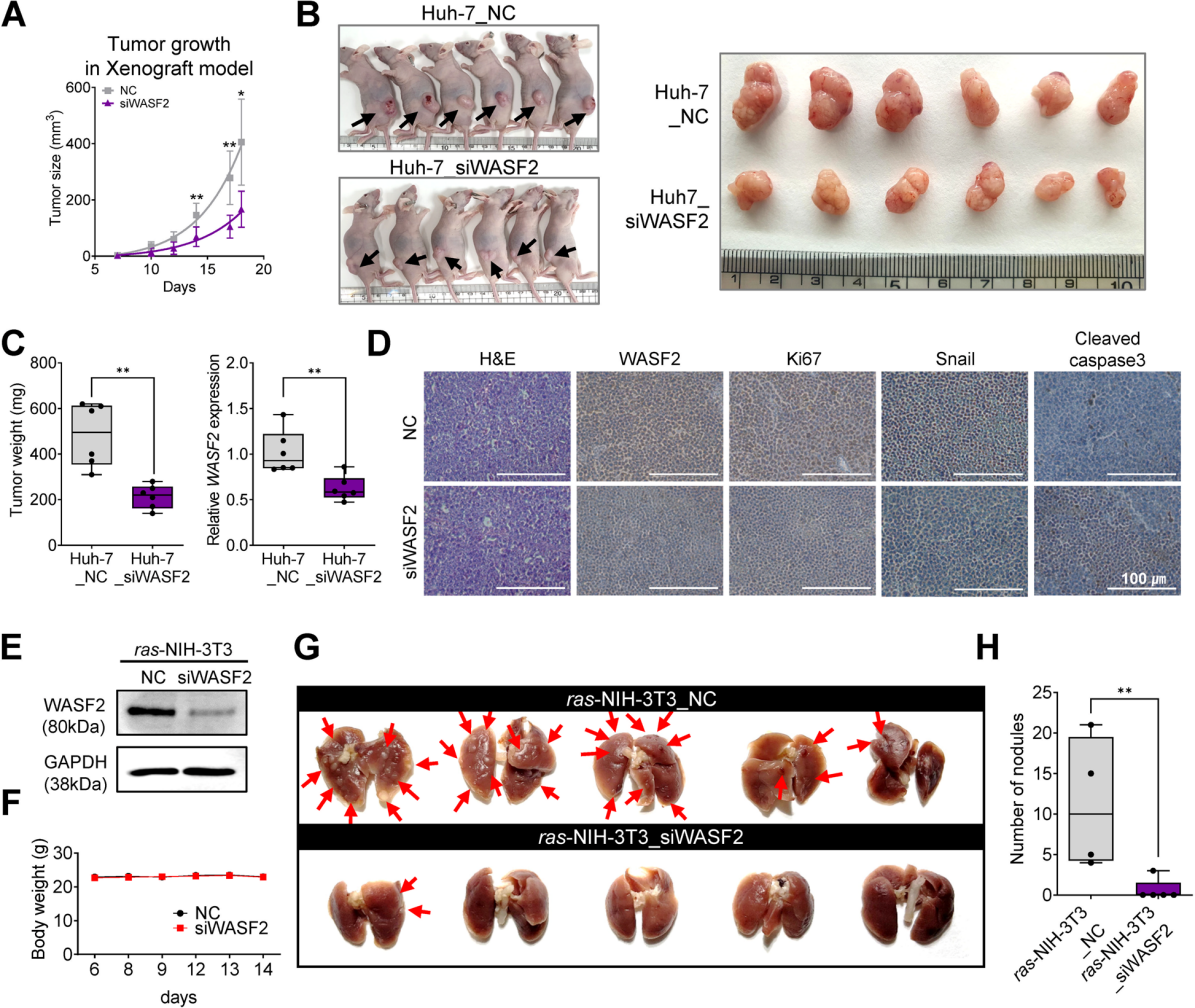
WASF2 suppression attenuates HCC tumorigenesis and metastatic in vivo. **A** Tumor growth in mice injected subcutaneously with Huh-7 cells inactivating WASF2 (*n* = 6 per group; unpaired *t*-test). **B** Mouse images (left) and mass images of xenograft mouse model established with Huh-7 cells transfected with negative control siRNA and siWASF2 (right). The arrows indicate tumor mass. **C** Box plot of tumor weight in two groups (left) and box plot of *WASF2* expression in xenograft tumor tissues assayed using qRT-PCR (right, *n* = 6 per group; unpaired *t*-test). **D** Representative images of H&E and IHC staining for WASF2, Ki-67, Snail, and cleaved caspase-3 in xenograft tumors derived from negative-control siRNA (NC) or siWASF2 transfected Huh-7 cells. Scale bar = 100 μm. **E** The efficiency of siWASF2 in *ras*-NIH-3 T3 cells and **F** body weight among the two groups of mice (*n* = 5 per group; unpaired *t*-test). **G** Representative in vivo images of nodules (arrow) 14 days after injection of transfected *ras*-transformed NIH-3 T3 cells with negative control siRNA (*n* = 5) or siWASF2 (*n* = 5) using a lung metastasis mouse model. **H** The number of nodules on the surface of the mouse lungs was counted. *P < 0.05; **P < 0.01. Data were shown as mean ± SEM

To gain further insights into the regulatory effect of *WASF2*, we analyzed *WASF2* expression in mouse and rat genomic datasets and assessed the tumor-suppressive effect of *WASF2* knockdown in vivo. *WASF2* expression was increased in early and advanced HCC samples from both diethylnitrosamine (DEN)-induced mouse and rat HCC models from the gene expression omnibus database compared to that in the corresponding control liver tissues (Fig. S7). To validate the tumor suppressive effects of *WASF2* knockdown in vivo, we subcutaneously injected siWASF2-transfected Huh-7 cells into BALB/c female nude mice. The overall rate of tumor growth was significantly lower in the *WASF2*-knockdown (Huh-7_siWASF2) group than that in the negative control (Huh-7_NC) group (Fig. 4A); in addition, the tumor size was lower (Fig. 4B). The average tumor volume and *WASF2* expression were significantly lower in the Huh-7_siWASF2 group than that in the Huh-7_NC group (Fig. 4C). We performed IHC analysis using anti-WASF2, anti-Ki67, anti-Snail, and anti-cleaved caspase-3 in xenograft tumor sections from the Huh-7_siWASF2 and Huh-7_NC groups. To avoid quantifying any non-tumoral areas, the xenograft sections were stained using H&E and WASF2. The expression levels of cytosolic WASF2, nuclear Ki67, and the cytosolic and nuclear Snail were decreased in the Huh-7_siWASF2 group, while the expression levels of the cytosolic and nuclear cleaved caspase-3 increased (Fig. 4D). To investigate the effects of *WASF2* on HCC cells metastasis, in vivo metastasis assays were performed. First, we confirmed the efficiency of the siRNAs targeting *WASF2* in *ras*-NIH-3 T3 cells (Fig. 4E); we then injected siWASF2-transfected *ras*-NIH-3 T3 cells into athymic female nude mice through tail veins. There were no distinct changes in the body weight between the two groups (Fig. 4F) during the 14 days of cell inoculation. Lung metastases were clearly observed in the *ras*-NIH-3T3_NC group, but not in the *ras*-NIH-3T3_siWASF2 group at the time of sacrifice (Fig. 4G). In addition, the number of lung nodules in the *ras*-NIH-3T3_siWASF2 group was significantly decreased than that in the *ras*-NIH-3T3_NC group (Fig. 4H). Together, these in vivo analyses indicate that WASF2 suppression attenuates the in vivo tumorigenic and metastatic potential of HCC.

### WASF2 promoter hypomethylation causes overexpression in hepatocarcinogenesis and is associated with poor prognosis in patients with HCC

Next, we attempted to identify the regulatory mechanism of *WASF2* activation. In the TCGA_LIHC dataset, *WASF2* methylation correlated significantly and negatively with *WASF2* expression in the tumor group (Pearson’s correlation coefficient *r* = ⎻0.33, *P* < 0.0001; Additional file 2: Fig. S8A. In addition, *WASF2* hypomethylation was higher in the tumor group compared to that in the normal group, in 44 matched pairs of HCC patients from TCGA_LIHC data set (Fig. S8B). Therefore, we analyzed the correlation between *WASF2* expression and the DNA methylation of CpG islands in the 5′ promoter region using MEXPRESS. Regression analysis indicated a significant correlation between *WASF2* expression and the methylation of five CpG sites (cg20745431, cg16406658, cg03436453, cg00307483, and cg24162579; Additional file 2: Fig. S8C), among which cg24162579 was significantly hypomethylated in the tumor samples of 44 matched pairs of patients with HCC from TCGA_LIHC (Additional file 2: Fig. S8D).

**Fig. 5:**
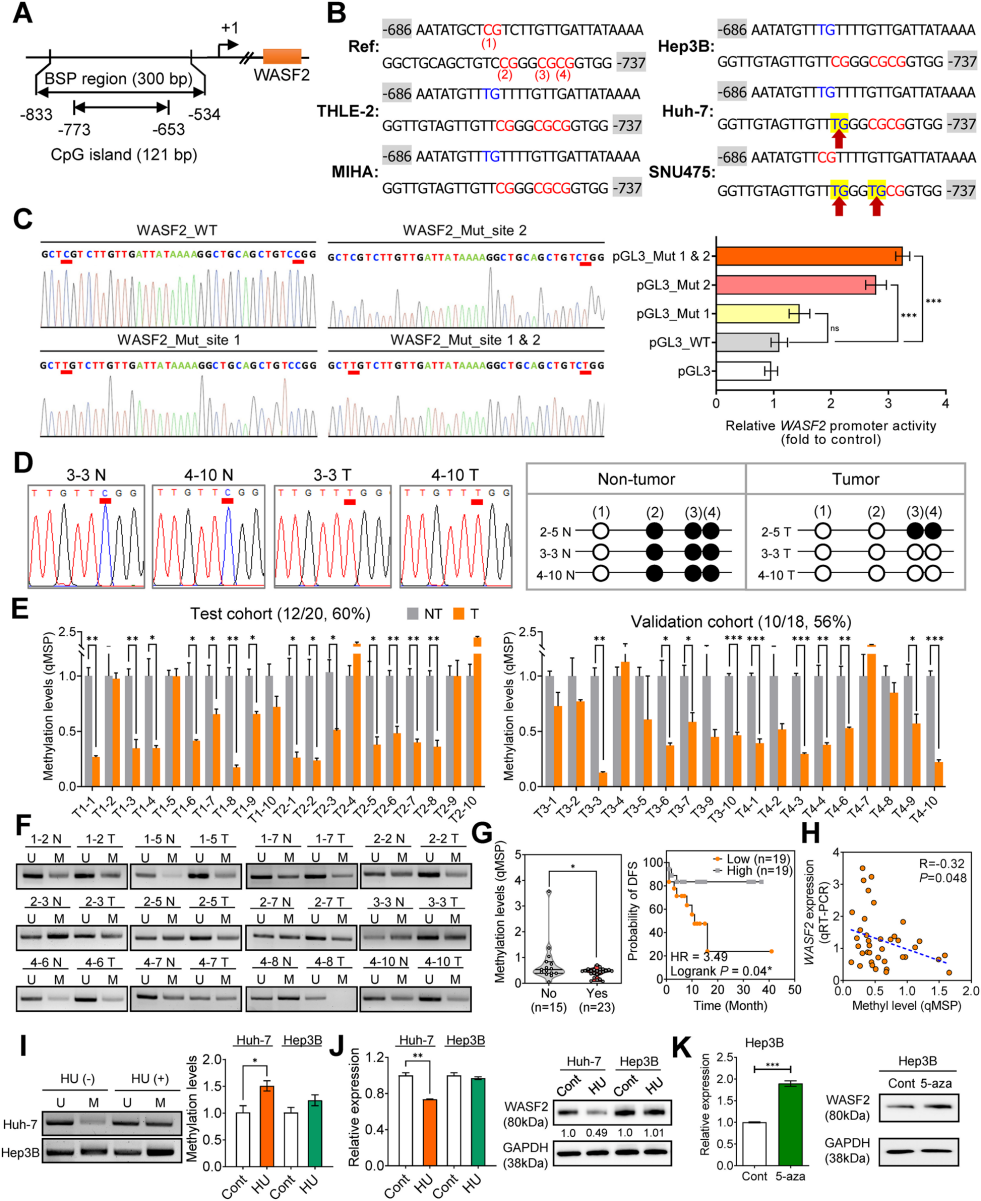
Oncogenic *WASF2* activation mechanism by methylation in HCC. **A** The genomic locale of *WASF2* and the CpG island in its promoter region. Arrow indicates transcriptional start site. The CpG island region was predicted using MethPrimer and the regions analyzed by BSP are indicated. **B** Sequence detail of the BSP regions (− 833 to − 534 bp) in the *WASF2* gene promoter. CpG dinucleotides (CG) in this region are indicated in red capitals and numbered 1–4. Methylation status of CpGs in the *WASF2* promoter region in HCC cell lines. Red arrows: sites specifically unmethylated in HCC cells . **C** BSP image for four luciferase reporter vectors, containing wild type, site 1 mutation only, site 2 mutation only, and site 1 & 2 mutation, respectively. Red underscore indicates mutation site (left). Promoter activity of wild-type WASF2 (pGL3_WT) and mutated WASF2 (pGL3_Mut 1 and 2), analyzed by luciferase assay (right). **D** Representative direct sequencing of the BSP image (left) and dot plots of methylation status of CpG site 2 (− 726 bp) in human HCC tissues (right). White and black dots indicate unmethylated and methylated CpGs, respectively. **E** Bar chart of CpG methylation in the *WASF2* promoter in the test cohort (top; *n* = 20) and validation cohort (bottom; *n* = 18) determined by MSP in paired tumor (T) and non-tumor (NT) tissues. **F** Representative gel images of MSP results. Primers are specific for unmethylated (U) or methylated (M) DNA. **G** Violin plot (left) of *WASF2* methylation based on the vascular invasion status of the test and validation cohorts [without invasiveness, No (*n* = 15); with invasiveness, Yes (*n* = 23); * *P* < 0.05]. Disease-free survival of HCC patients with low or high *WASF2* DNA methylation (right). *P* values determined by the log-rank test. **H** Correlation of *WASF2* methylation status and its expression in 66 HCC tissues. **I** Methylation status (left) and levels (right) of WASF2 in Huh-7 and Hep3B cells with/without HU treatment. **J** WASF2 mRNA (left) and protein (right) expression in Huh-7 and Hep3B cells with/without HU treatment. **K** *WASF2* mRNA and protein expression in Hep3B cells with/without 5-aza treatment. *P < 0.05; **P < 0.01; ***P < 0.001

Consequently, we assessed the clinical relevance of *WASF2* methylation status in patients with HCC using the cg24162579 site. We performed BSP and MSP analysis on the *WASF2* promoter in immortalized normal liver hepatocytes (THLE-2 and MIHA) and HCC (Hep3B,Huh-7 and SNU475) cell lines targeting the region including the cg24162579 site (− 833 to − 534 bp relative to the transcriptional start site, Fig. 5A). Site 2 of CpG sites of cg24162579 in the *WASF2* promoter region was unmethylated in Huh-7 and SNU475 cells, but it was methylated in the other cell lines (Fig. 5B, Additional file 2: Fig. S9A).

To further confirm the functional consequences of demethylation on the gene expression of CpG site 1 (− 694 bp) and 2 (− 726 bp) located in the *WASF2* promoter, a point mutation of cytosine to thymine was inserted at CpG sites 1 and 2 located in the *WASF2* promoter (Fig. 5C, left). The wild-type *WASF2* (pGL3_WT) and mutated *WASF2* (pGL3_Mut 1 and 2) were transiently transfected into Huh-7 cells. Wild-type *WASF2* had no significant effect on expression compared to that in the control group. Mutation of cytosine at CpG site 1 (− 694 bp) of *WASF2* with thymine did not have a significant influence on the expression; however, the cytosine-to-thymine conversion of CpG site 2 (− 726 bp) of *WASF2* significantly increased the expression compared to that with wild-type *WASF2* (Fig. 5C). The mutation of CpG sites 1 and 2 showed a synergistic effect on the increase in *WASF2* expression (Fig. 5C, right). Therefore, the methylation pattern of CpG site 2 (− 726 bp) located in the *WASF2* promoter plays an important role in the regulation of WASF2 expression. Subsequently, we randomly selected paired human HCC tissues and confirmed that CpG site 2 (− 726 bp) was methylated in the non-tumor group and unmethylated in the tumor group (Fig. 5D). We analyzed *WASF2* promoter methylation levels in paired human HCC tissues using qMSP; 60 and 56% of the tumor tissues in the test (*n* = 20) and validation (*n* = 18) cohorts, respectively, were hypomethylated compared to non-tumor tissues (Fig. 5E, F). We decided to assess the clinical relevance of *WASF2* methylation status in these 38 patients with HCC. *WASF2* methylation was lower in HCC tissues with vascular invasiveness than that in those without the invasiveness (Fig. 5G, left); the DFS of HCC patients with cg24162579 hypomethylation was lower than that of patients with hypermethylation (Fig. 5G, right). The *WASF2* methylation status significantly and negatively correlated with *WASF2* expression in these 38 HCC tissues (Pearson’s correlation coefficient *r* = ⎻0.32, *P =* 0.048; Fig. 5H).

To determine whether WASF2 is regulated by DNA methylation-dependent epigenetic mechanisms, we treated Hep3B (CpG site 2 methylated cell line) and Huh-7 cells (CpG site 2 unmethylated cell line) with the hyper-methylating agent HU or DNA methylation inhibitor 5-aza. We measured *WASF2* promoter DNA methylation using MSP and WASF2 expression using qRT-PCR and western blot. HU treatment increased DNA methylation and decreased WASF2 expression at the RNA and protein levels in Huh-7 cells compared to that in untreated cells; however, it did not affect the levels in Hep3B cells (Fig. 5I, J). Treatment with 5-aza increased the RNA and protein levels of WASF2 in Hep3B cells (Fig. 5K). Therefore, WASF2 expression is regulated via a DNA methylation-mediated epigenetic mechanism. To investigate the effect of DNA methyltransferases (DNMT)-dependent methylation on WASF2 in HCC cells, the expression levels of DNMT1, DNMT3a, and DNMT3b were measured following the knockdown of each DNMT in Huh-7 and Hep3B. The mRNA level of each *DNMT* in the Huh-7 and Hep3B cells was significantly decreased compared to that in the negative control (Additional file 2: Fig. S9B, top); the expression of *WASF2* was upregulated (Additional file 2: Fig. S9B, bottom).

**Fig. 6:**
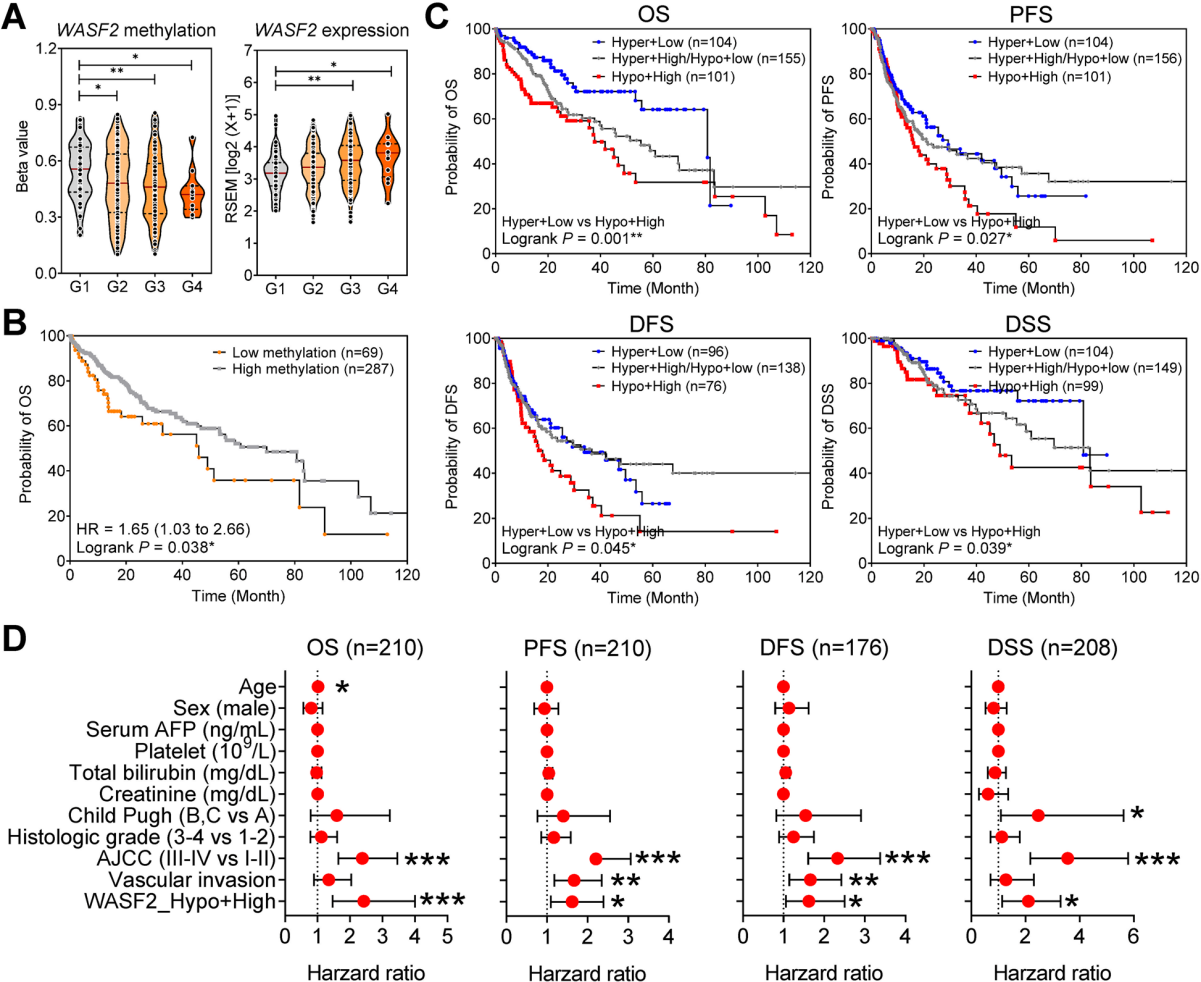
Clinical association between *WASF2* methylation and overexpression. **A** Differences in cg24162579 CpG site methylation in the *WASF2* promoter region according to HCC tumor grade (left). Differences in *WASF2* expression according to HCC tumor grade (right). **B** Overall survival curves according to cg24162579 CpG site methylation in TCGA_LIHC dataset. **C** Overall survival, progression-free survival, disease-free survival, and disease-specific survival curves according to the level of *WASF2* methylation and expression in the TCGA_LIHC dataset. **D** Forest plot of univariate Cox regression analyses of clinical parameters on overall survival, progression-free survival, disease-free survival, and disease-specific survival *P < 0.05; **P < 0.01; ***P < 0.001. HCC differentiation was defined using the Edmondson grade scale (grade 1, G1; grade 2, G2; grade 3, G3; and grade 4, G4)

Finally, we evaluated the clinical association between *WASF2* methylation and overexpression in TCGA_LIHC dataset. cg24162579 CpG methylation in the *WASF2* promoter was significantly downregulated according to HCC tumor stage (Fig. 6A, left); however, *WASF2* expression was significantly upregulated (Fig. 6A, right). *WASF2* methylation and expression were significantly correlated with HCC tumor differentiation; therefore, we assessed their prognostic effects in patients with HCC according to *WASF2* methylation and expression level, using the median beta value or median *WASF2* expression as the threshold. Patients in the low methylation group had a shorter OS than those in the high methylation group (log-rank *p* = 0.038, HR = 1.65; Fig. 6B). Patients with high-methylation (Hyper) and low-expression (Low) showed the most favorable OS, progression-free survival (PFS), DFS, and disease-specific survival (DSS; *P* = 0.001, *P* = 0.027, *P* = 0.045, and *P* = 0.039, respectively; Fig. 6C, D). Multivariate analysis demonstrated that *WASF2* with hypomethylation and with high expression was an independent poor prognostic factor for OS in HCC (Additional file 1: Table S6, S7, S8 and S9). Therefore, *WASF2* methylation and expression appear to have a significant correlation with the prognosis of patients with HCC.

## Discussion

Through an in-depth analysis of public omics databases and validation experiments with HCC cell lines, xenograft mouse model, and human samples, this study performed comprehensive functional characterization of WASF2 in HCC. cg24162579 CpG site 2 methylation in the promoter could be an important regulatory mechanism of WASF2.

We attempted to detect autoantibodies for tumor-associated antigens and identify hepatocarcinogenesis biomarkers using 22 K protein chip analysis in patients with and without HCC [14, 15]. WASF2 autoantibodies were detected in plasma at the time of diagnosis in all five patients with early HCC, and in the plasma of four patients 1 year before diagnosis. Patients with high *WASF2* expression in the TCGA_LIHC dataset had a lower OS rate than those with low *WASF2* expression. Consequently, we decided to characterize the role of WASF2 in HCC and its regulatory mechanism.

WASF2 plays a key role in cell motility by relaying Rac activation to induce Arp2/3-mediated actin polymerization [16], while playing critical roles in cell movement and angiogenesis during embryogenesis [5, 17]. In addition, WASF2 overexpression is clinically relevant in cancers such as melanoma, pancreatic cancer, and breast cancer as well as in liver metastasis in colorectal cancer [6, 7, 18–20]. WASF2 mRNA and protein levels are higher in the HCC tissues than that in the paired surrounding non-tumor tissues (*n* = 31); they are significantly associated with aggressive tumor behavior such as multiple tumor number and vessel invasion. Patients with high WASF2 expression in HCC tissues (≥ 26% cancer cells) have a lower OS than those with low expression (≤ 25% cancer cells) [8]. The role of WASF2 in the phenotype of HCC remains largely unclear; therefore, this study provides a comprehensive understanding of WASF2 expression in HCC tissues and its correlation with clinical outcomes. It demonstrates the effects of WASF2 suppression in HCC in vitro and in vivo. *WASF2* expression was increased in the HCC tissues from six public omics databases and increased significantly according to the progression of liver disease. Patients with high *WASF2* expression had a lower OS, DSS, and PFS than those with low *WASF2* expression. We validated WASF2 mRNA and protein overexpression in 54.5% (36/66) and 72.7% (16/22) of the patients with HCC from our own cohort, before examining the biological functions of WASF2 in HCC using in vitro and in vivo mouse models. WASF2 inactivation decreased the viability, growth, proliferation, migration, and invasion of Huh-7 and SNU475 HCC cells, by inducing G2/M phase arrest, provoking HCC cell death, inhibiting EMT, and modulating actin polymerization. WASF2 inactivation resulted in tumor suppressive effects in the xenograft mouse model. Therefore, WASF2 suppression affects the cell cycle, cell death, EMT regulation, and actin cytoskeleton, which are all critical factors in tumorigenesis [21–23].

microRNA-1253 is a novel epigenetic regulator of WASF2 in human umbilical vein endothelial cells and human aortic endothelial cells [9]; however, there are no studies on the epigenetic mechanisms that regulate WASF2 expression in cancer. In addition, studies on WASF2 regulation in cancer cells are limited. There is a negative correlation between *WASF2* methylation status and mRNA expression in TCGA_LIHC dataset. cg24162579 was the only significantly hypomethylated CpG island in the *WASF2* 5′ promoter region in HCC samples from TCGA_LIHC dataset. We also confirmed CpG site 2 (− 726 bp) of cg24162579 located in the *WASF2* promoter plays an important role in the regulation of WASF2 expression in vitro*.* Clinically, cg24162579 methylation decreased and *WASF2* expression increased according to HCC tumor differentiation grade. Patients with low cg24162579 methylation showed a significantly lower OS than those with high methylation. Together, these findings suggest that patients with hypermethylation and low expression of *WASF2* have the most favorable OS.

## Conclusions

In conclusion, *WASF2* is overexpressed in HCC and its expression correlates with poor patient prognosis. *WASF2* suppression decreased HCC cell growth, migration, and invasion, by inducing G2/M phase cell cycle arrest and HCC cell death, inhibiting EMT, and hindering actin polymerization; cg24162579 methylation is a key epigenetic mechanism that regulates *WASF2* expression and regulates prognosis, suggesting that *WASF2* could be a potential therapeutic target for HCC.

## Supplementary Information

**Additional file 1: Table S1.** Demographics and clinical characteristics of patients. **Table S2.** Primer sequences. **Table S3.** Primary antibodies. **Table S4.** Secondary antibodies. **Table S5**. ROC results of 47 autoantibodies. **Table S6.** Univariate and multivariate Cox regression analyses of factors associated with overall survival. **Table S7.** Univariate and multivariate Cox regression analyses of factors associated with progression-free survival. **Table S8.** Univariate and multivariate Cox regression analyses of factors associated with disease-free survival. **Table S9.** Univariate and multivariate Cox regression analyses of factors associated with disease-specific survival.

**Additional file 2: Fig. S1.** A *WASF2* expression in matched pairs of patients with HCC from TCGA_LIHC, ICGC_LIRI, and GSE77314 datasets. B Differential expression of *WASF2* in human hepatocarcinogenesis in GSE6764, GSE12443, and GSE114564 datasets (one-way ANOVA, post hoc comparisons, Tukey’s test). C Representative images of WASF2 expression in HCC tissues and D proportion of patients with different WASF2 immunostaining intensity in HCC specimens based on Human Protein Atlas (HPA) data. Scale bar = 200 μm. E Kaplan–Meier survival analyses of *WASF2* expression in TCGA_LIHC datasets for overall survival (left), disease-specific survival (middle), and progression-free survival (right; log-rank test *P < 0.05; **P < 0.01; ***P < 0.001). Abbreviations: HCC, hepatocellular carcinoma; TCGA_LIHC, The Cancer Genome Atlas liver hepatocellular carcinoma project; ICGC_LIRI, International Cancer Genome Consortium liver cancer RIKEN Japan. **Fig. S2.** A Efficiency of three different siWASF2 in Huh-7 and SNU475 cells assessed using MTT assay (top) and western blot analysis (bottom). siWASF2 #1 was used for the subsequent experiments due to its better efficiency. B After transfection with negative control siRNA (NC) or siWASF2 for 48 and 72 h, the knockdown efficiencies in HCC cells were measured using qRT-PCR (top) and western blot analysis (bottom). **Fig. S3.** A Cluster analysis was performed on 1354 gene signatures that correlated highly with *WASF2* expression (*P* < 0.001, *r* > 0.3 or *r* < − 0.3) in TCGA_LIHC dataset. Patients were divided into *WASF2* high or *WASF2* low groups. B Kaplan–Meier analysis of overall survival (top) and disease-free survival (bottom; ***P < 0.0001; log-rank test). C Bar chart of the top 20 gene set lists enriched in the *WASF2*-associated gene signature reported by The Molecular Signatures Database (MSigDB) (FDR *q* < 0.01). D Gene Set Enrichment Analysis (GSEA) plots for the HCC-related gene sets derived from the WASF2 signature. Y-axis represents ES, X-axis represents genes (vertical black lines) in the gene sets. Significance was determined from the nominal *P* values (≤ 0.05) and false discovery rate (≤ 0.25) from GSEA. **Fig. S4.** Cell death after transfection with siWASF2. Fragmented nuclei stained with Hoechst33342/PI indicate apoptotic bodies (yellow arrows) or necrotic cells (white arrows). Representative fluorescence images. Scale bar = 100 μm. **Fig. S5.** Correlation analysis between *WASF2* and *SNAI1* mRNA expression in non-tumor liver tissues (*n* = 50, Pearson’s correlation coefficient, *r* = 0.08, *P* = 0.55, top) and HCC tissues (*n* = 371, Pearson’s correlation coefficient, *r* = 0.26, *P* < 0.0001, bottom) from TCGA_LIHC dataset. **Fig. S6.** Network analysis of actin cytoskeleton signaling pathways related with WASF2 using IPA. **Fig. S7.** Differential expression of *WASF2* in a mouse model of DEN-induced hepatocarcinogenesis (left: GSE93392) and a rat model (right: GSE102418) (one-way ANOVA, post hoc comparisons, Tukey’s test). **Fig. S8.** A Correlation analysis between WASF2 mRNA expression and methylation in TCGA_LIHC dataset (*n* = 360, Pearson’s correlation coefficient, *r* = − 0.33, ***P < 0.0001). B Density plot of the methylation status in the non-tumor (NT) and tumor (T) groups. Methylation distribution across all sites. X-axis represents methylation level as mean β-values. Y-axis represents relative density. C DNA methylation of CpG islands in the 5′ promoter region corresponding to *WASF2* expression. Right: Pearson’s correlation coefficient *r* and *p* values for Wilcoxon rank-sum test between each parameter and *WASF2* expression. Red = significant difference. D *WASF2* methylation level of the five significant CpG sites (cg20745431, cg16406658, cg03436453, cg00307483, and cg24162579) in the matched pairs of HCC patients from TCGA_LIHC. **Fig. S9.** A Direct sequencing of TA-cloned BSP products was used to determine the methylation status of each CpG island in immortalized hepatocyte cell lines (THLE-2 and MIHA) and HCC cell lines (Hep3B, Huh-7, and SNU475). B The efficiency of siRNAs on the silencing of DNMT1, DNMT3a, and DNMT3b in Huh-7 and Hep3B cells assessed using qRT-PCR for 48 h (top) and after transfection with siDNMT1 or siDNMT3a or siDNMT3b for 48 h, *WASF2* mRNA levels in HCC cells were evaluated through qRT-PCR (bottom).

## Data Availability

All data that support the findings of this study are available from the corresponding authors upon reasonable request.

## Abbreviations

Arp2/3: Actin-related protein 2/3
ANOVA: Analysis of variance
AUC: Area under the curve
BSP: Bisulfite sequencing polymerase chain reaction
Catholic_LIHC: The Catholic University of Korea’s liver hepatocellular carcinoma project
Cdc42: Cell division control protein 42 homolog
DEN: Diethylnitrosamine
DFS: Disease-free survival
DNMT: DNA methyltransferase
DSS: Disease-specific survival
EMT: Epithelial-mesenchymal transition
ES: Enrichment score
EX-WASF2: Overexpression WASF2
GAPDH: Glyceraldehyde 3-phosphate dehydrogenase
GSEA: Gene set enrichment analysis
H&E: Hematoxylin and eosin
HCC: Hepatocellular carcinoma
HU: Hydroxyurea
HPA: Human protein atlas
ICGC_LIRI: International Cancer Genome Consortium liver cancer RIKEN Japan
IHC: Immunohistochemistry
IPA: Ingenuity pathway analysis
Mut: Mutation
MSigDB: The Molecular Signatures Database
MSP: Methylation-specific polymerase chain reaction
MTT: 3-(4,5-Dimethylthiazol-2-yl)-2,5-diphenyltetrazolium bromide
OS: Overall survival
PARP: Poly ADP-ribose polymerase
PBS: Phosphate-buffered saline
PFS: Progression-free survival
PI: Propidium iodide
qMSP: Quantitative methylation-specific polymerase chain reaction
qRT-PCR: Quantitative real-time polymerase chain reaction
Rac1: Ras-related C3 botulinum toxin substrate 1
ROC: Receiver operating characteristic
SEM: Standard error of mean
siNC: Negative control small interfering RNA
siRNA: Small interfering RNA
siWASF2: WASF2-targeting small interfering RNA
TCGA_LIHC: The Cancer Genome Atlas liver hepatocellular carcinoma project
TGF- β1: Transforming growth factor-β1
WASF2: Wiskott-Aldrich Syndrome Protein Family Member 2
WT: Wild type
ZO-1: Zonula occuludens-1
5-aza: 5-aza-2′-deoxycytidine
