# Supplementary material for: Hypomethylation-mediated upregulation of the WASF2 promoter region correlates with poor clinical outcomes in hepatocellular carcinoma
Source: J Exp Clin Cancer Res. 2022 Apr 28;41:158. doi: 10.1186/s13046-022-02365-7 (PMC9047373; doi:10.1186/s13046-022-02365-7)
Supplement: Supplementary file 1 — Additional file 1: Table S1. Demographics and clinical characteristics of patients. Table S2. Primer sequences. Table S3. Primary antibodies. Table S4. Secondary antibodies. Table S5. ROC results of 47 autoantibodies. Table S6. Univariate and multivariate Cox regression analyses of factors associated with overall survival. Table S7. Univariate and multivariate Cox regression analyses of factors associated with progression-free survival. Table S8. Univariate and multivariate Cox regression analyses of factors associated with disease-free survival. Table S9. Univariate and multivariate Cox regression analyses of factors associated with disease-specific survival. [file 13046_2022_2365_MOESM1_ESM.docx]

**Table S1.** Demographics and clinical characteristics of patients

| Characteristics | | *n* = 66 |
| --- | --- | --- |
| Age, years | | 56.4 (10.1) |
| Gender (male) | | 52 (78.8) |
| Etiology | |  |
|  | HBV | 60 (90.9) |
|  | HCV | 5 (7.6) |
|  | Alcohol | 1 (1.5) |
| Cirrhosis | | 26 (39.4) |
| Platelet, x 10^3^/μL | | 176.4 (68.7) |
| Albumin, g/dL | | 4.5 (0.7) |
| Total bilirubin, mg/dL | | 0.87 (1.1) |
| Creatinine, mg/dL | | 0.86 (0.19) |
| Sodium, mmol/L | | 139.4 (2.1) |
| AST, IU/L | | 52.3 (82.3) |
| ALT, IU/L | | 46.7 (57.6) |
| AFP (>200 ng/mL) | | 15 (23.1) |
| PIVKA-II (>40 mAU/mL) | | 40 (65.6) |
| Macrovascular invasion | | 18 (27.3) |
| Lymph node metastasis | | 2 (3.0) |
| Distant metastasis | | 2 (3.0) |
| BCLC stage | |  |
|  | 0 | 16 (24.2) |
|  | A | 31 (47.0) |
|  | B | 2 (3.0) |
|  | C | 17 (25.8) |
|  | D | 0 (0.0) |
| mUICC stage | |  |
|  | I | 18 (27.3) |
|  | II | 28 (42.4) |
|  | III | 15 (22.7) |
|  | IVA | 2 (3.0) |
|  | IVB | 3 (4.5) |

Data were expressed as number (%) or mean (standard deviation).

**Table S2.** Primer sequences

| **Primer** | | **Nucleotide Sequence** |
| --- | --- | --- |
| *WASF2* | Forward | 5'-AGCCTTCAGAAGTTCCACCA-3' |
|  | Reverse | 5'-CTGCAGCATCTTCTCC TTCC-3' |
| *DNMT1* | Forward | 5’-CCCTACCGAATTGGCCGGAT-3’ |
|  | Reverse | 5’-GCAGGTTGATGTCTGCGTGG-3’ |
| *DNMT3a* | Forward | 5’-GTCAAAACCCCACCTGGAGC-3’ |
|  | Reverse | 5’-ACATCACATTCCAGGGGCCG-3’ |
| *DNMT3b* | Forward | 5’-ACCACCTGCTGAATTACTCACGC-3’ |
|  | Reverse | 5’-GATGGCATCAATCATCACTGGATT-3’ |
| *HMBS* | Forward | 5’- GGAGGGCAGAAGGAAGAAAACAG-3’ |
|  | Reverse | 5’- CACTGTCCGTCTGTATGCGAG-3’ |
| *GAPDH* | Forward | 5'-AGTATGACAACAGCCTCAAG-3' |
|  | Reverse | 5'-TCATGAGTCCTTCCACGATA-3' |
| *WASF2*_bisulfite | Forward | 5’-TTTATGTATGAGTGGGGAATATGTT-3’ |
|  | Reverse | 5’-TCAACCTCCCAAAATACTAAAATTAC-3’ |
| *WASF2*_M | Forward | 5’-AGTAGTCGTTTGTGTTTTATATGCG-3’ |
|  | Reverse | 5’-CCAAATTAATCAAACTAATCTCGAA-3’ |
| *WASF2*_UM | Forward | 5’-GTAGTTGTTTGTGTTTTATATGTGG-3’ |
|  | Reverse | 5’-CCAAATTAATCAAACTAATCTCAAA-3’ |

**Table S3.** Primary antibodies

|  | **Antibody** | | **Species** | **Dilution** | **Manufacturer** |
| --- | --- | --- | --- | --- | --- |
| **Human IHC** | | | | | |
|  | WASF2 | | Rabbit | 1:500 | Atlas Antibodies |
|  | CCT5 | | Rabbit | 1:200 | Atlas Antibodies |
|  | PGK1 | | Rabbit | 1:50 | Atlas Antibodies |
|  | SF3B4 | | Rabbit | 1:50 | Atlas Antibodies |
| **Mouse IHC** | | | | | |
|  | WASF2 | | Rabbit | 1:500 | Atlas Antibodies |
|  | Snail | | Rabbit | 1:50 | Cell Signaling Technology |
|  | Cleaved Caspase-3 | | Rabbit | 1:400 | Cell Signaling Technology |
|  | Ki-67 | | Mouse | 1:50 | Santa Cruz Biotechnology |
| **Immunofluorescence** | | | | | |
|  | F-actin | | Mouse | 1:200 | Abcam |
|  | **Antibody** | **Molecular weight (kDa)** | **Species** | **Dilution** | **Manufacturer** |
| **Western blot** | | | | | |
|  | WASF2 | 80 | Rabbit | 1:1000 | Cell Signaling Technology |
|  | FLAG-probe | 48 | Mouse | 1:1000 | Santa Cruz Biotechnology |
|  | p53 | 53 | Mouse | 1:1000 | Santa Cruz Biotechnology |
|  | CDK4 | 30 | Mouse | 1:1000 | Cell Signaling Technology |
|  | CDK6 | 36 | Mouse | 1:2000 | Cell Signaling Technology |
|  | p-Wee1 | 95 | Rabbit | 1:1000 | Cell Signaling Technology |
|  | PARP | 116 | Rabbit | 1:1000 | Cell Signaling Technology |
|  | Cleaved PARP | 89 | Rabbit | 1:1000 | Cell Signaling Technology |
|  | Caspase-3 | 35 | Rabbit | 1:1000 | Cell Signaling Technology |
|  | Cleaved Caspase-3 | 17 | Rabbit | 1:1000 | Cell Signaling Technology |
|  | Caspase-9 | 49 | Mouse | 1:1000 | Cell Signaling Technology |
|  | Cleaved Caspase-9 | 37 | Rabbit | 1:1000 | Cell Signaling Technology |
|  | ZO-1 | 195 | Mouse | 1:1000 | Thermo Fisher Scientific |
|  | E-cadherin | 120 | Mouse | 1:1000 | BD bioscience |
|  | Fibronectin | 220 | Mouse | 1:1000 | Santa Cruz Biotechnology |
|  | N-cadherin | 130 | Mouse | 1:2000 | BD bioscience |
|  | Vimentin | 54 | Rabbit | 1:5000 | GeneTex |
|  | Snail+Slug | 29–68 | Mouse | 1:250 | Abcam |
|  | Rac/cdc42 | 21 | Rabbit | 1:1000 | Cell Signaling Technology |
|  | p-Rac/cdc42 | 28 | Rabbit | 1:1000 | Cell Signaling Technology |
|  | Arp2 | 44 | Rabbit | 1:1000 | Cell Signaling Technology |
|  | Arp3 | 47 | Rabbit | 1:1000 | Cell Signaling Technology |
|  | F-actin | 42 | Mouse | 1:500 | Abcam |
|  | GAPDH | 38 | Mouse | 1:1000 | Santa Cruz Biotechnology |

**Table S4.** Secondary antibodies

| **Secondary antibody** | | **Dilution** | **Manufacturer** |
| --- | --- | --- | --- |
| **Immunofluorescence** | |  |  |
|  | Donkey anti-Mouse IgG (AlexaFluor-488 conjugated) | 1:500 | Invitrogen |
| **Western blot** | |  |  |
|  | Goat anti-Rabbit IgG H&L (HRP) | 1:3 000 | Bio-Rad Laboratories |
|  | Goat anti-Mouse IgG H&L (HRP) | 1:3 000 | Bio-Rad Laboratories |

**Table S5**. ROC results of 47 autoantibodies

| **Gene** | **AUC** | **SE** | **P value** | **95% CI** |
| --- | --- | --- | --- | --- |
| **WASF2** | **0.883** | **0.076** | **0.001** | **0.734-1** |
| **ASAP2** | **0.85** | **0.084** | **0.004** | **0.686-1** |
| **CUTA** | **0.85** | **0.084** | **0.004** | **0.686-1** |
| **PGK1** | **0.833** | **0.091** | **0.006** | **0.655-1** |
| **SF384** | **0.8** | **0.096** | **0.013** | **0.612-0.988** |
| **Nc2b** | **0.8** | **0.089** | **0.013** | **0.626-0.974** |
| **PUF60** | **0.767** | **0.101** | **0.027** | **0.569-0.964** |
| **STAT4** | **0.767** | **0.101** | **0.027** | **0.569-0.964** |
| **CCT5** | **0.767** | **0.095** | **0.027** | **0.581-0.952** |
| **ACBD6** | **0.733** | **0.105** | **0.052** | **0.528-0.938** |
| **BRD9** | **0.733** | **0.1** | **0.052** | **0.538-0.929** |
| **DR1** | **0.733** | **0.1** | **0.052** | **0.538-0.929** |
| **MED4** | **0.733** | **0.1** | **0.052** | **0.538-0.929** |
| **TMEM163** | **0.733** | **0.1** | **0.052** | **0.538-0.929** |
| **TSNAXIP1** | **0.733** | **0.1** | **0.052** | **0.538-0.929** |
| **CHD4** | **0.7** | **0.104** | **0.096** | **0.496-0.904** |
| **DMP1** | **0.7** | **0.104** | **0.096** | **0.496-0.904** |
| **KLK5** | **0.7** | **0.104** | **0.096** | **0.496-0.904** |
| **OGFR** | **0.7** | **0.104** | **0.096** | **0.496-0.904** |
| **P3H4** | **0.7** | **0.104** | **0.096** | **0.496-0.904** |
| **RNF25** | **0.7** | **0.104** | **0.096** | **0.496-0.904** |
| **SFT2D2** | **0.7** | **0.104** | **0.096** | **0.496-0.904** |
| **TTC1** | **0.7** | **0.104** | **0.096** | **0.496-0.904** |
| GPD1 | 0.683 | 0.108 | 0.127 | 0.472-0.895 |
| ARHGEF16 | 0.667 | 0.108 | 0.166 | 0.456-0.878 |
| DRICH1 | 0.667 | 0.108 | 0.166 | 0.456-0.878 |
| EHD2 | 0.667 | 0.108 | 0.166 | 0.456-0.878 |
| HAO2 | 0.667 | 0.108 | 0.166 | 0.456-0.878 |
| MTMR3 | 0.667 | 0.108 | 0.166 | 0.456-0.878 |
| NAP1L5 | 0.667 | 0.108 | 0.166 | 0.456-0.878 |
| PDIA2 | 0.667 | 0.108 | 0.166 | 0.456-0.878 |
| PPP2R1B | 0.667 | 0.108 | 0.166 | 0.456-0.878 |
| ZCCHC7 | 0.667 | 0.108 | 0.166 | 0.456-0.878 |
| APH1A | 0.633 | 0.111 | 0.267 | 0.416-0.851 |
| ATP5B | 0.633 | 0.111 | 0.267 | 0.416-0.851 |
| COQ9 | 0.633 | 0.111 | 0.267 | 0.416-0.851 |
| DPCR1 | 0.633 | 0.111 | 0.267 | 0.416-0.851 |
| IZUMO4 | 0.633 | 0.111 | 0.267 | 0.416-0.851 |
| MAP4 | 0.633 | 0.111 | 0.267 | 0.416-0.851 |
| NFIX | 0.633 | 0.111 | 0.267 | 0.416-0.851 |
| NM_024269.4_frag | 0.633 | 0.111 | 0.267 | 0.416-0.851 |
| PDZRN4 | 0.633 | 0.111 | 0.267 | 0.416-0.851 |
| PRH1 | 0.633 | 0.111 | 0.267 | 0.416-0.851 |
| RHOXF2 | 0.633 | 0.111 | 0.267 | 0.416-0.851 |
| TMPRSS2 | 0.633 | 0.111 | 0.267 | 0.416-0.851 |
| TMPRSS4 | 0.633 | 0.111 | 0.267 | 0.416-0.851 |
| TCOF1 | 0.617 | 0.114 | 0.332 | 0.394-0.839 |

**Table S6.** Univariate and multivariate Cox regression analyses of factors associated with overall survival

|  | OS | | | | | |
| --- | --- | --- | --- | --- | --- | --- |
|  | **Univariate** | | | **Multivariate** | | |
| Factor | **HR** | **95% CI** | ***P value*** | **HR** | **95% CI** | ***P value*** |
| Age | 1.015 | 1.001-1.029 | ***0.040*** | 1.013 | 1.994-1.032 | 0.189 |
| Sex (male) | 0.805 | 0.565-1.149 | 0.232 |  |  |  |
| Serum AFP  (ng/mL) | 1.000 | 1.000-1.000 | 0.433 |  |  |  |
| Platelet  (mg/dL) | 1.000 | 1.000-1.000 | 0.575 |  |  |  |
| Total bilirubin  (mg/mL) | 0.971 | 0.840-1.121 | 0.684 |  |  |  |
| Creatinine  (mg/dL) | 1.002 | 0.986-1.018 | 0.820 |  |  |  |
| Child Pugh  (B,C vs A) | 1.591 | 0.785-3.223 | 0.198 |  |  |  |
| Histologic grade  (3-4 vs 1-2) | 1.114 | 0.776-1.599 | 0.559 |  |  |  |
| AJCC  (III-IV vs I-II) | 2.378 | 1.637-3.454 | ***5.46E-06*** | 2.358 | 1.424-3.903 | ***0.001*** |
| Vascular invasion  (Yes vs No) | 1.343 | 0.886-2.034 | 0.164 |  |  |  |
| WASF2 methylation [Hypo_High(n=106) vs Hyper_Low (n=104)] | 2.420 | 1.465-3.999 | ***0.001*** | 2.052 | 1.213-3.472 | ***0.007*** |

**Table S7.** Univariate and multivariate Cox regression analyses of factors associated with progression-free survival

|  | PFS | | | | | |
| --- | --- | --- | --- | --- | --- | --- |
|  | **Univariate** | | | **Multivariate** | | |
| Factor | **HR** | **95% CI** | ***P value*** | **HR** | **95% CI** | ***P value*** |
| Age | 0.995 | 0.984-1.006 | 0.329 |  |  |  |
| Sex (male) | 0.940 | 0.688-1.283 | 0.694 |  |  |  |
| Serum AFP  (ng/mL) | 1.000 | 1.000-1.000 | 0.384 |  |  |  |
| Platelet  (mg/dL) | 1.000 | 1.000-1.000 | 0.743 |  |  |  |
| Total bilirubin  (mg/mL) | 1.043 | 0.955-1.140 | 0.348 |  |  |  |
| Creatinine  (mg/dL) | 1.001 | 0.986-1.016 | 0.871 |  |  |  |
| Child Pugh  (B,C vs A) | 1.400 | 0.766-2.556 | 0.274 |  |  |  |
| Histologic grade  (3-4 vs 1-2) | 1.166 | 0.859-1.583 | 0.324 |  |  |  |
| AJCC  (III-IV vs I-II) | 2.203 | 1.585-3.061 | ***2.58E-06*** | 1.807 | 1.085-3.008 | ***0.023*** |
| Vascular invasion  (Yes vs No) | 1.665 | 1.181-2.347 | ***0.004*** | 1.052 | 0.645-1.717 | 0.838 |
| WASF2 methylation [Hypo_High(n=106) vs Hyper_Low (n=104)] | 1.620 | 1.096-2.395 | ***0.017*** | 1.423 | 0.902-2.245 | 0.130 |

**Table S8.** Univariate and multivariate Cox regression analyses of factors associated with disease-free survival

|  | DFS | | | | | |
| --- | --- | --- | --- | --- | --- | --- |
|  | **Univariate** | | | **Multivariate** | | |
| Factor | **HR** | **95% CI** | ***P value*** | **HR** | **95% CI** | ***P value*** |
| Age | 0.996 | 0.983-1.008 | 0.510 |  |  |  |
| Sex (male) | 1.132 | 0.793-1.614 | 0.494 |  |  |  |
| Serum AFP  (ng/mL) | 1.000 | 1.000-1.000 | 0.426 |  |  |  |
| Platelet  (mg/dL) | 1.000 | 1.000-1.000 | 0.966 |  |  |  |
| Total bilirubin  (mg/mL) | 1.050 | 0.960-1.150 | 0.288 |  |  |  |
| Creatinine  (mg/dL) | 0.997 | 0.978-1.015 | 0.710 |  |  |  |
| Child Pugh  (B,C vs A) | 1.543 | 0.820-2.905 | 0.179 |  |  |  |
| Histologic grade  (3-4 vs 1-2) | 1.244 | 0.887-1.745 | 0.206 |  |  |  |
| AJCC  (III-IV vs I-II) | 2.330 | 1.607-3.377 | ***8.05E-06*** | 2.329 | 1.364-3.976 | ***0.002*** |
| Vascular invasion  (Yes vs No) | 1.661 | 1.141-2.418 | ***0.008*** | 1.058 | 0.632-1.773 | 0.830 |
| WASF2 methylation [Hypo_High(n=80) vs Hyper_Low (n=96)] | 1.623 | 1.055-2.499 | ***0.028*** | 1.267 | 0.777-2.064 | 0.342 |

**Table S9.** Univariate and multivariate Cox regression analyses of factors associated with disease-specific survival

|  | DSS | | | | | |
| --- | --- | --- | --- | --- | --- | --- |
|  | **Univariate** | | | **Multivariate** | | |
| Factor | **HR** | **95% CI** | ***P value*** | **HR** | **95% CI** | ***P value*** |
| Age | 1.007 | 0.990-1.025 | 0.428 |  |  |  |
| Sex (male) | 0.821 | 0.520-1.297 | 0.398 |  |  |  |
| Serum AFP  (ng/mL) | 1.000 | 1.000-1.000 | 0.463 |  |  |  |
| Platelet  (mg/dL) | 1.000 | 1.000-1.000 | 0.305 |  |  |  |
| Total bilirubin  (mg/mL) | 0.876 | 0.602-1.276 | 0.491 |  |  |  |
| Creatinine  (mg/dL) | 0.616 | 0.279-1.360 | 0.231 |  |  |  |
| Child Pugh  (B,C vs A) | 2.470 | 1.084-5.626 | ***0.031*** | 5.293 | 1.795-15.608 | ***0.003*** |
| Histologic grade  (3-4 vs 1-2) | 1.128 | 0.704-1.790 | 0.627 |  |  |  |
| AJCC  (III-IV vs I-II) | 3.556 | 2.182-5.794 | ***3.53E-07*** | 2.075 | 0.861-5.002 | 0.104 |
| Vascular invasion  (Yes vs No) | 1.277 | 0.707-2.307 | 0.418 |  |  |  |
| WASF2 methylation [Hypo_High(n=104) vs Hyper_Low (n=104)] | 2.106 | 1.130-3.295 | ***0.019*** | 1.121 | 0.451-2.786 | 0.805 |
