# Supplementary material for: Hypomethylation-mediated upregulation of the WASF2 promoter region correlates with poor clinical outcomes in hepatocellular carcinoma
Source: J Exp Clin Cancer Res. 2022 Apr 28;41:158. doi: 10.1186/s13046-022-02365-7 (PMC9047373; doi:10.1186/s13046-022-02365-7)
Supplement: Supplementary file 2 — Additional file 2: Fig. S1. A WASF2 expression in matched pairs of patients with HCC from TCGA_LIHC, ICGC_LIRI, and GSE77314 datasets. B Differential expression of WASF2 in human hepatocarcinogenesis in GSE6764, GSE12443, and GSE114564 datasets (one-way ANOVA, post hoc comparisons, Tukey’s test). C Representative images of WASF2 expression in HCC tissues and D proportion of patients with different WASF2 immunostaining intensity in HCC specimens based on Human Protein Atlas (HPA) data. Scale bar = 200 μm. E Kaplan–Meier survival analyses of WASF2 expression in TCGA_LIHC datasets for overall survival (left), disease-specific survival (middle), and progression-free survival (right; log-rank test *P < 0.05; **P < 0.01; ***P < 0.001). Abbreviations: HCC, hepatocellular carcinoma; TCGA_LIHC, The Cancer Genome Atlas liver hepatocellular carcinoma project; ICGC_LIRI, International Cancer Genome Consortium liver cancer RIKEN Japan. Fig. S2. A Efficiency of three different siWASF2 in Huh-7 and SNU475 cells assessed using MTT assay (top) and western blot analysis (bottom). siWASF2 #1 was used for the subsequent experiments due to its better efficiency. B After transfection with negative control siRNA (NC) or siWASF2 for 48 and 72 h, the knockdown efficiencies in HCC cells were measured using qRT-PCR (top) and western blot analysis (bottom). Fig. S3. A Cluster analysis was performed on 1354 gene signatures that correlated highly with WASF2 expression (P < 0.001, r > 0.3 or r < − 0.3) in TCGA_LIHC dataset. Patients were divided into WASF2 high or WASF2 low groups. B Kaplan–Meier analysis of overall survival (top) and disease-free survival (bottom; ***P < 0.0001; log-rank test). C Bar chart of the top 20 gene set lists enriched in the WASF2-associated gene signature reported by The Molecular Signatures Database (MSigDB) (FDR q < 0.01). D Gene Set Enrichment Analysis (GSEA) plots for the HCC-related gene sets derived from the WASF2 signature. Y-axis represents ES, X-axis represent [file 13046_2022_2365_MOESM2_ESM.docx]

**
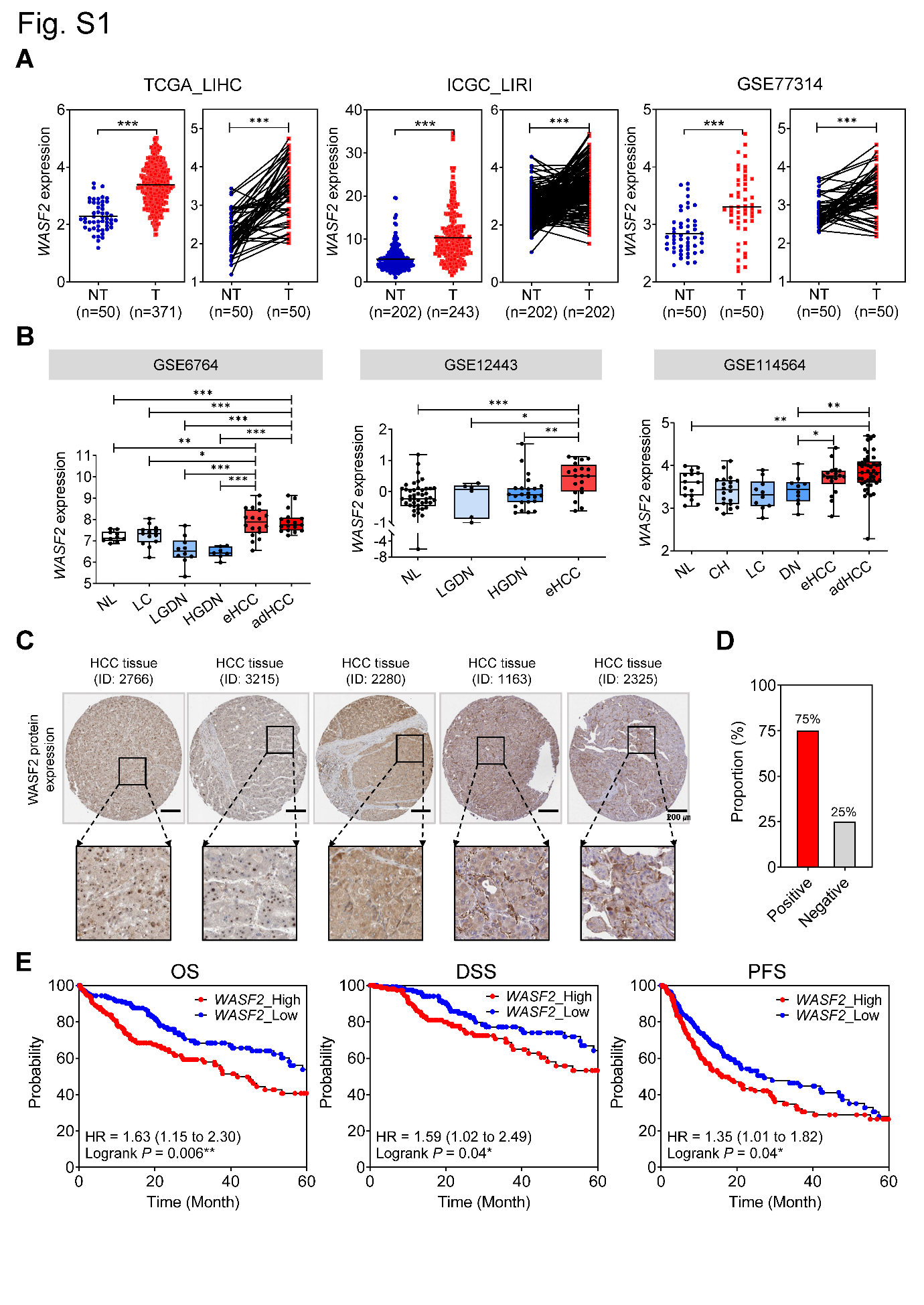
**

**Fig. S1 A** *WASF2* expression in matched pairs of patients with HCC from TCGA_LIHC, ICGC_LIRI, and GSE77314 datasets. **B** Differential expression of *WASF2* in human hepatocarcinogenesis in GSE6764, GSE12443, and GSE114564 datasets (one-way ANOVA, *post hoc* comparisons, Tukey's test). **C** Representative images of WASF2 expression in HCC tissues and **D** proportion of patients with different WASF2 immunostaining intensity in HCC specimens based on Human Protein Atlas (HPA) data. Scale bar = 200 µm. **E** Kaplan–Meier survival analyses of *WASF2* expression in TCGA_LIHC datasets for overall survival (left), disease-specific survival (middle), and progression-free survival (right; log-rank test **P* < 0.05; ***P* < 0.01; ****P* < 0.001).


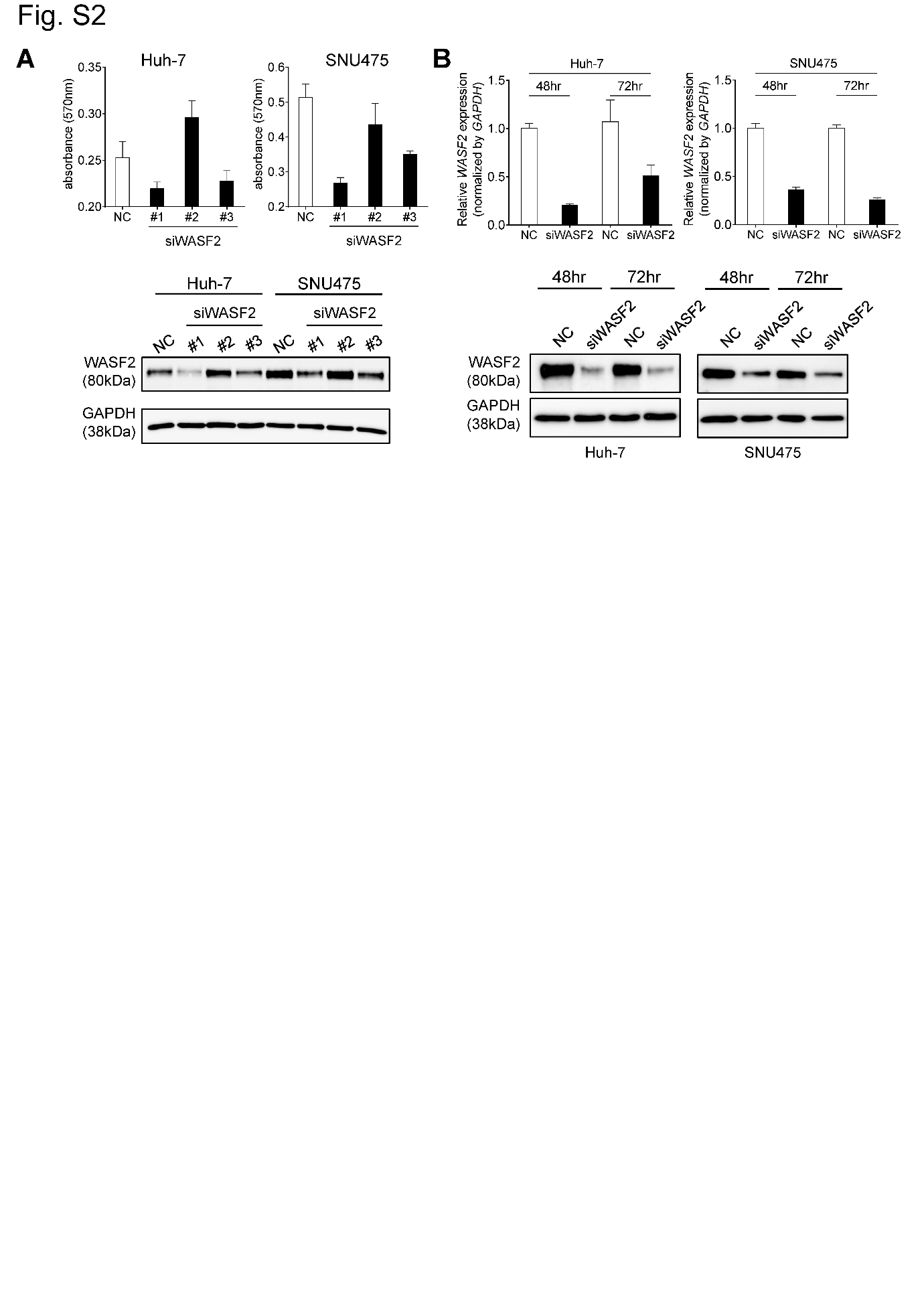


**Fig. S2 A** Efficiency of three different siWASF2 in Huh-7 and SNU475 cells assessed using MTT assay (top) and western blot analysis (bottom). siWASF2 #1 was used for the subsequent experiments due to its better efficiency. **B** After transfection with negative control siRNA (NC) or siWASF2 for 48 and 72 h, the knockdown efficiencies in HCC cells were measured using qRT-PCR (top) and western blot analysis (bottom).


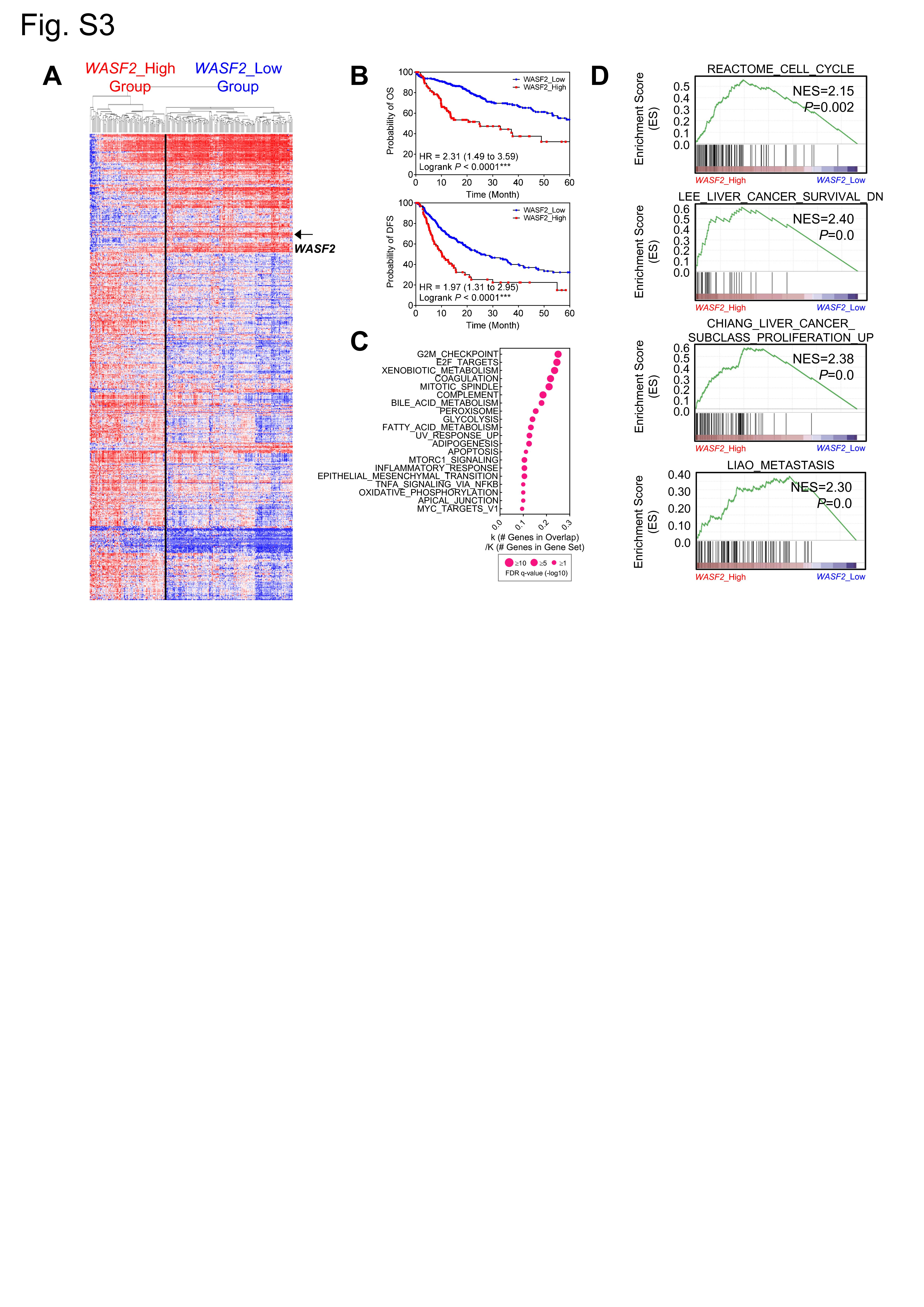


**Fig. S3 A** Cluster analysis was performed on 1,354 gene signatures that correlated highly with *WASF2* expression (*P* < 0.001, *r* > 0.3 or *r* < −0.3) in TCGA_LIHC dataset. Patients were divided into *WASF2* high or *WASF2* low groups. **B** Kaplan–Meier analysis of overall survival (top) and disease‐free survival (bottom; ****P* < 0.0001; log-rank test). **C** Bar chart of the top 20 gene set lists enriched in the *WASF2*-associated gene signature reported by The Molecular Signatures Database (MSigDB) (FDR *q* < 0.01). **D** Gene Set Enrichment Analysis (GSEA) plots for the HCC-related gene sets derived from the WASF2 signature. Y-axis represents ES, X-axis represents genes (vertical black lines) in the gene sets. Significance was determined from the nominal *P* values (≤ 0.05) and false discovery rate (≤ 0.25) from GSEA.


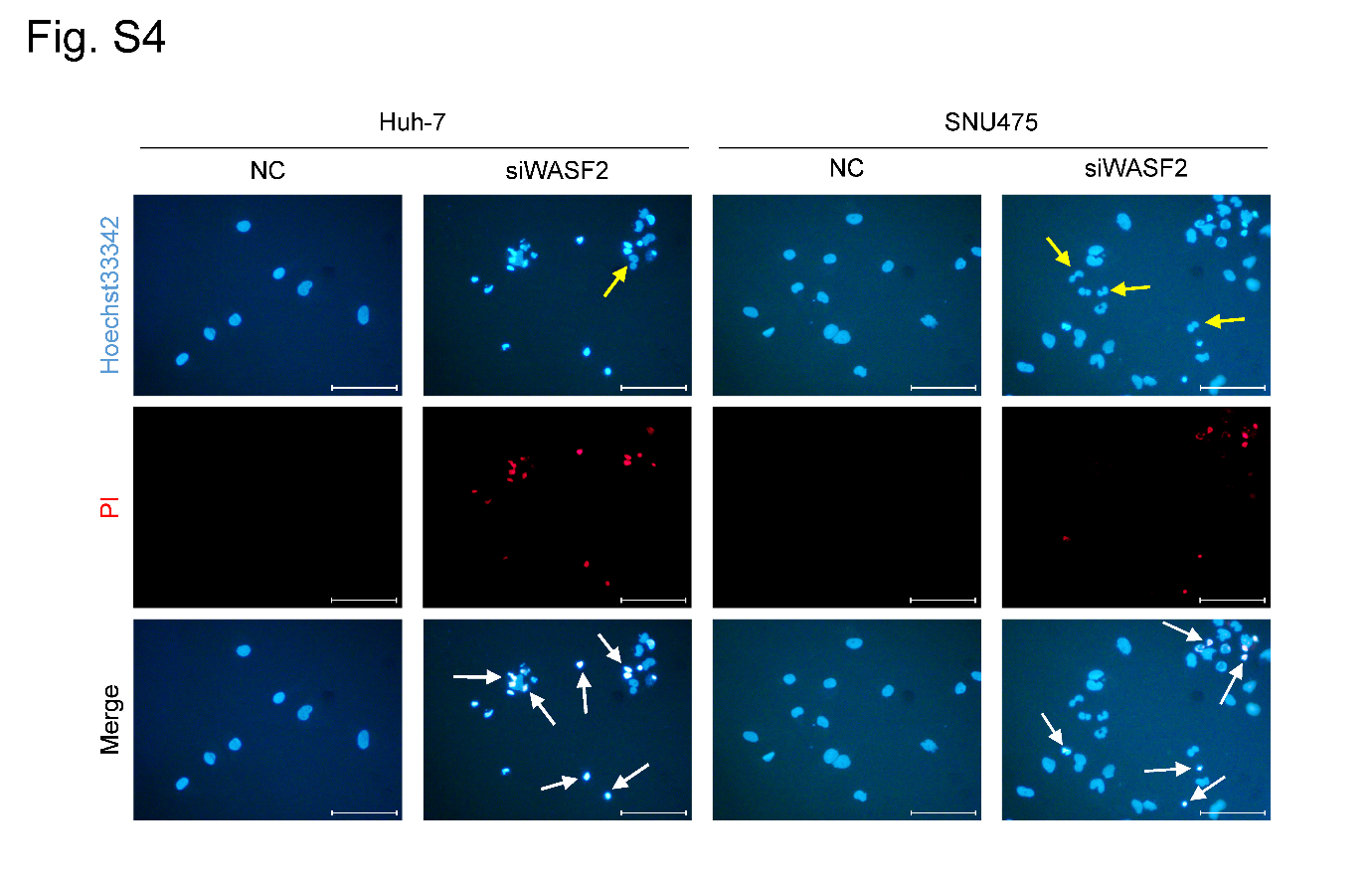


**Fig. S4** Cell death after transfection with siWASF2. Fragmented nuclei stained with Hoechst33342/PI indicate apoptotic bodies (yellow arrows) or necrotic cells (white arrows). Representative fluorescence images. Scale bar = 100 µm.

**
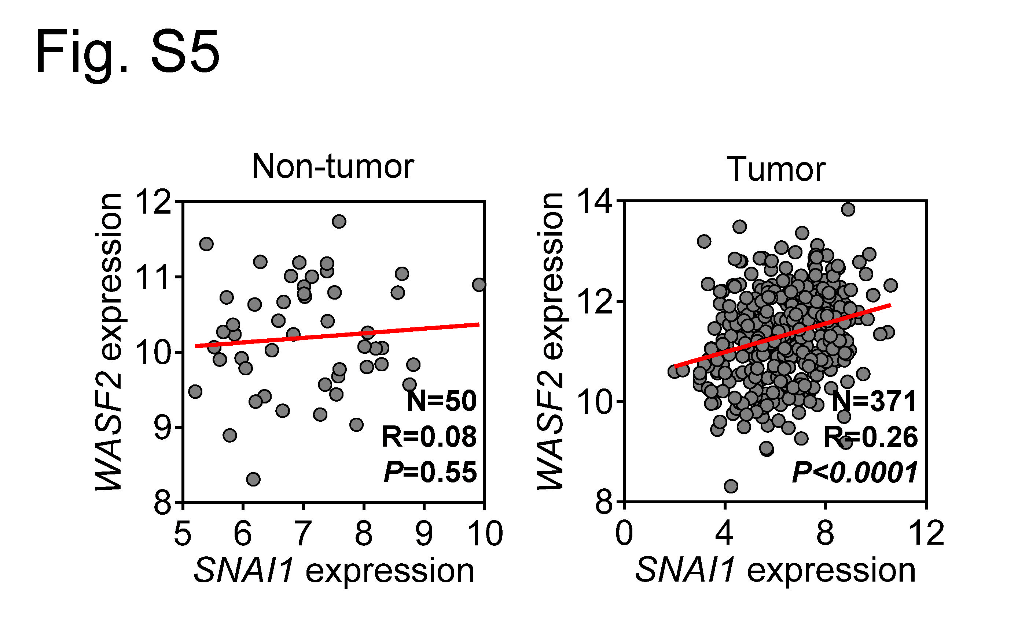
**

**Fig. S5** Correlation analysis between *WASF2* and *SNAI1* mRNA expression in non-tumor liver tissues (*n* = 50, Pearson’s correlation coefficient, *r* = 0.08, *P* = 0.55, top) and HCC tissues (*n* = 371, Pearson’s correlation coefficient, *r* = 0.26, *P* < 0.0001, bottom) from TCGA_LIHC dataset.

**
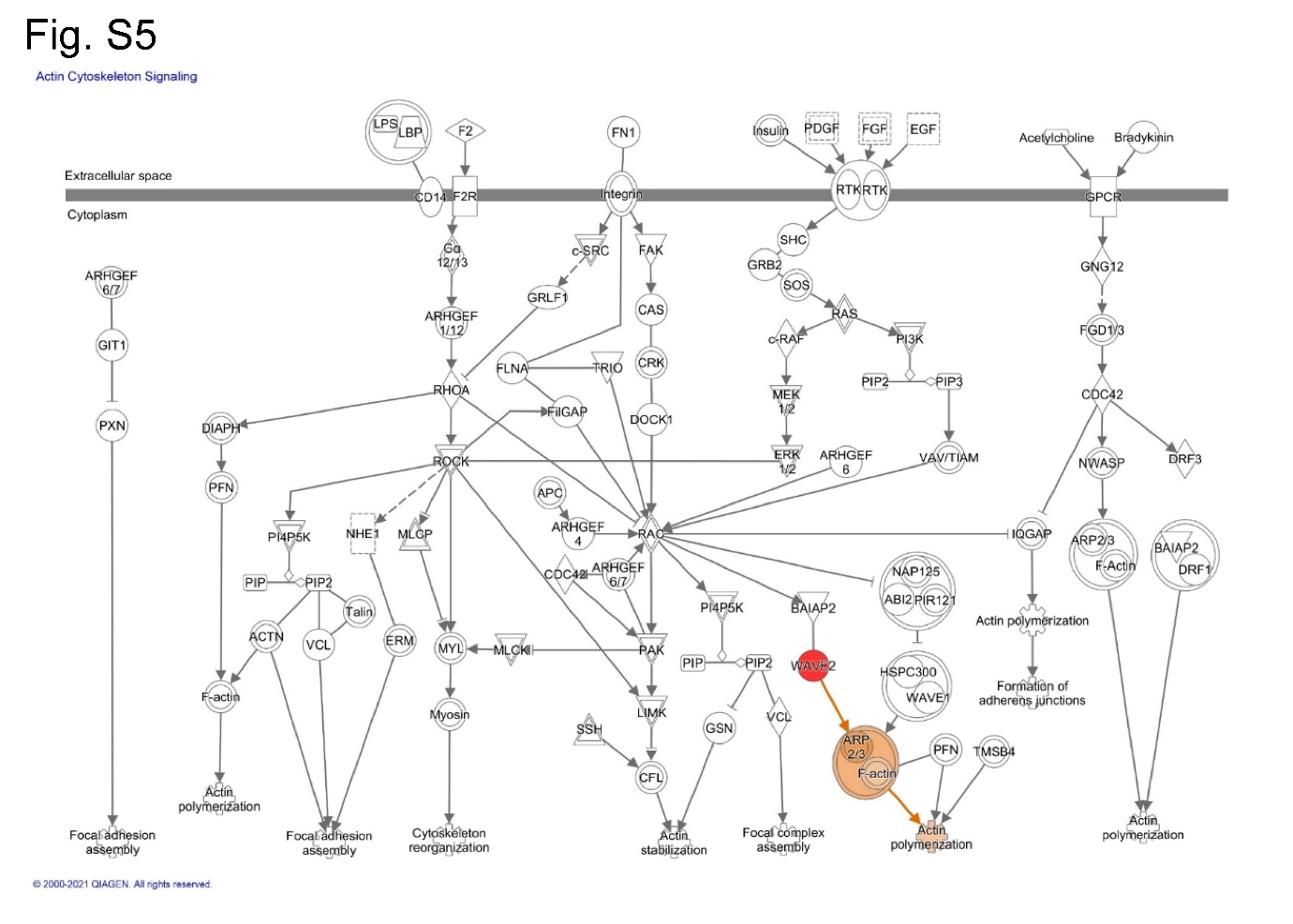
**

**Fig. S6** Network analysis of actin cytoskeleton signaling pathways related with WASF2 using IPA.

**
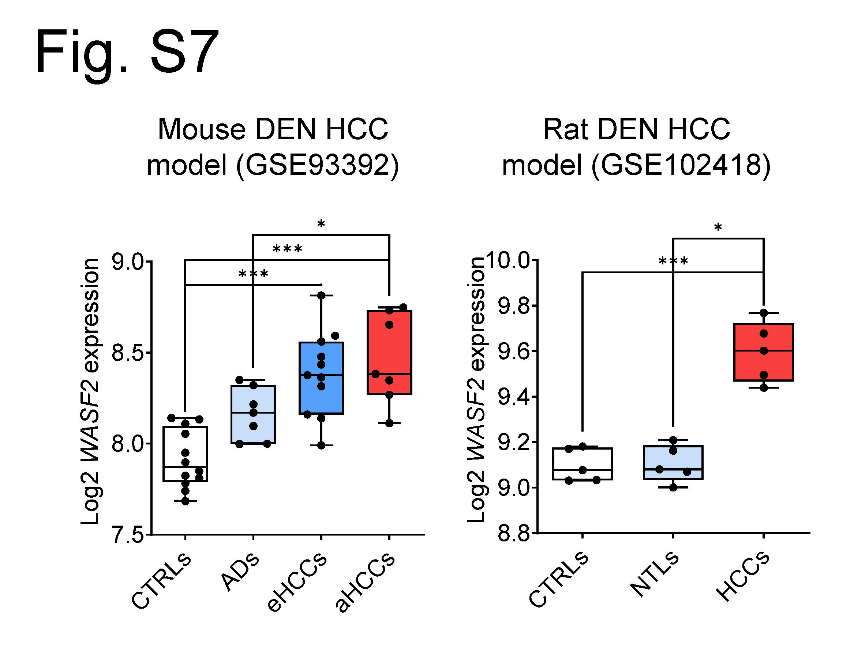
**

**Fig. S7** Differential expression of *WASF2* in a mouse model of DEN-induced hepatocarcinogenesis (left: GSE93392) and a rat model (right: GSE102418) (one-way ANOVA, *post hoc* comparisons, Tukey's test).


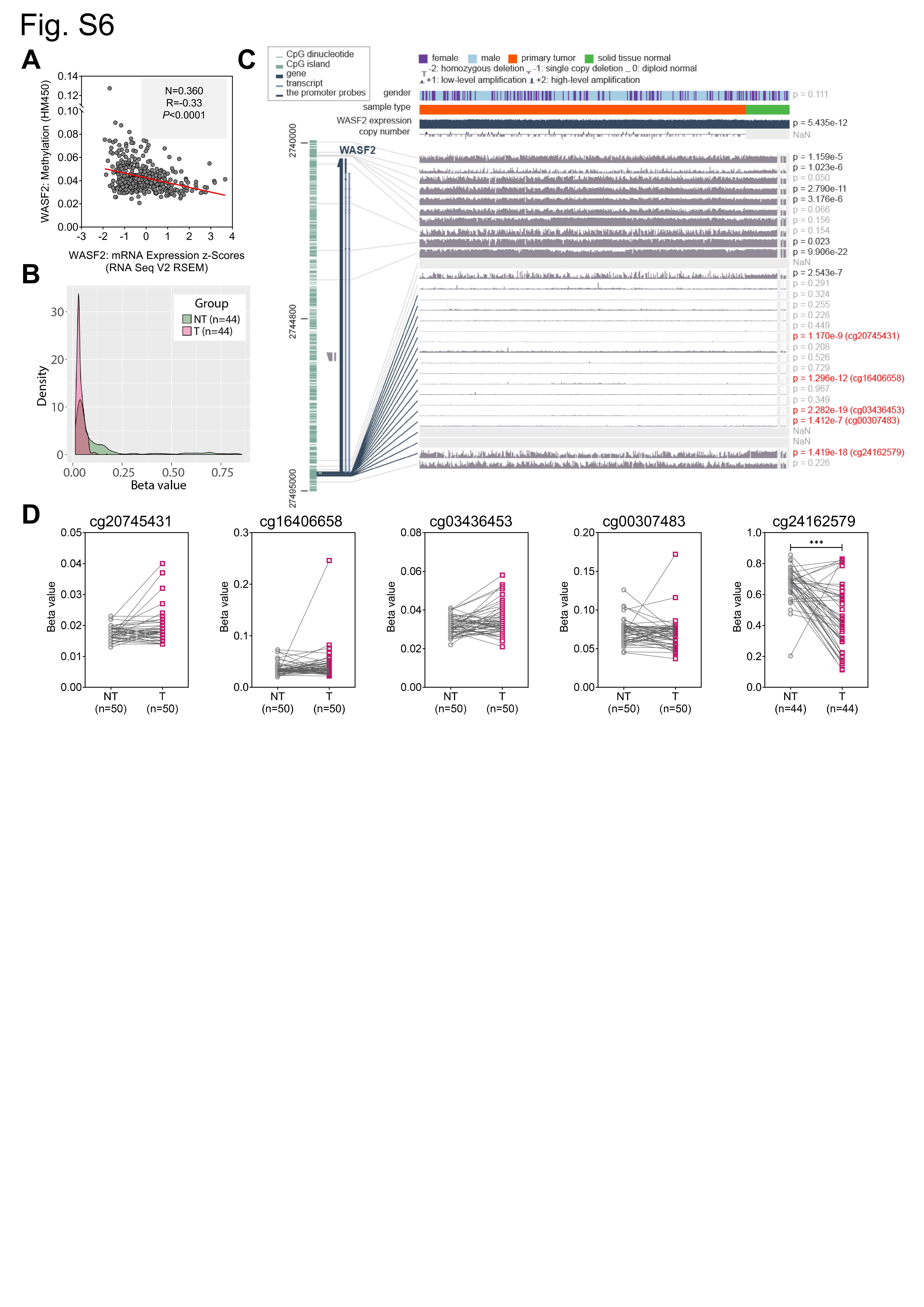


**Fig. S8 A** Correlation analysis between WASF2 mRNA expression and methylation in TCGA_LIHC dataset (*n* = 360, Pearson’s correlation coefficient, *r* = −0.33, ****P* < 0.0001). **B** Density plot of the methylation status in the non-tumor (NT) and tumor (T) groups. Methylation distribution across all sites. X-axis represents methylation level as mean β-values. Y-axis represents relative density. **C** DNA methylation of CpG islands in the 5′ promoter region corresponding to *WASF2* expression. Right: Pearson’s correlation coefficient *r* and *p* values for Wilcoxon rank-sum test between each parameter and *WASF2* expression. Red = significant difference. **D** *WASF2* methylation level of the five significant CpG sites (cg20745431, cg16406658, cg03436453, cg00307483, and cg24162579) in the matched pairs of HCC patients from TCGA_LIHC.


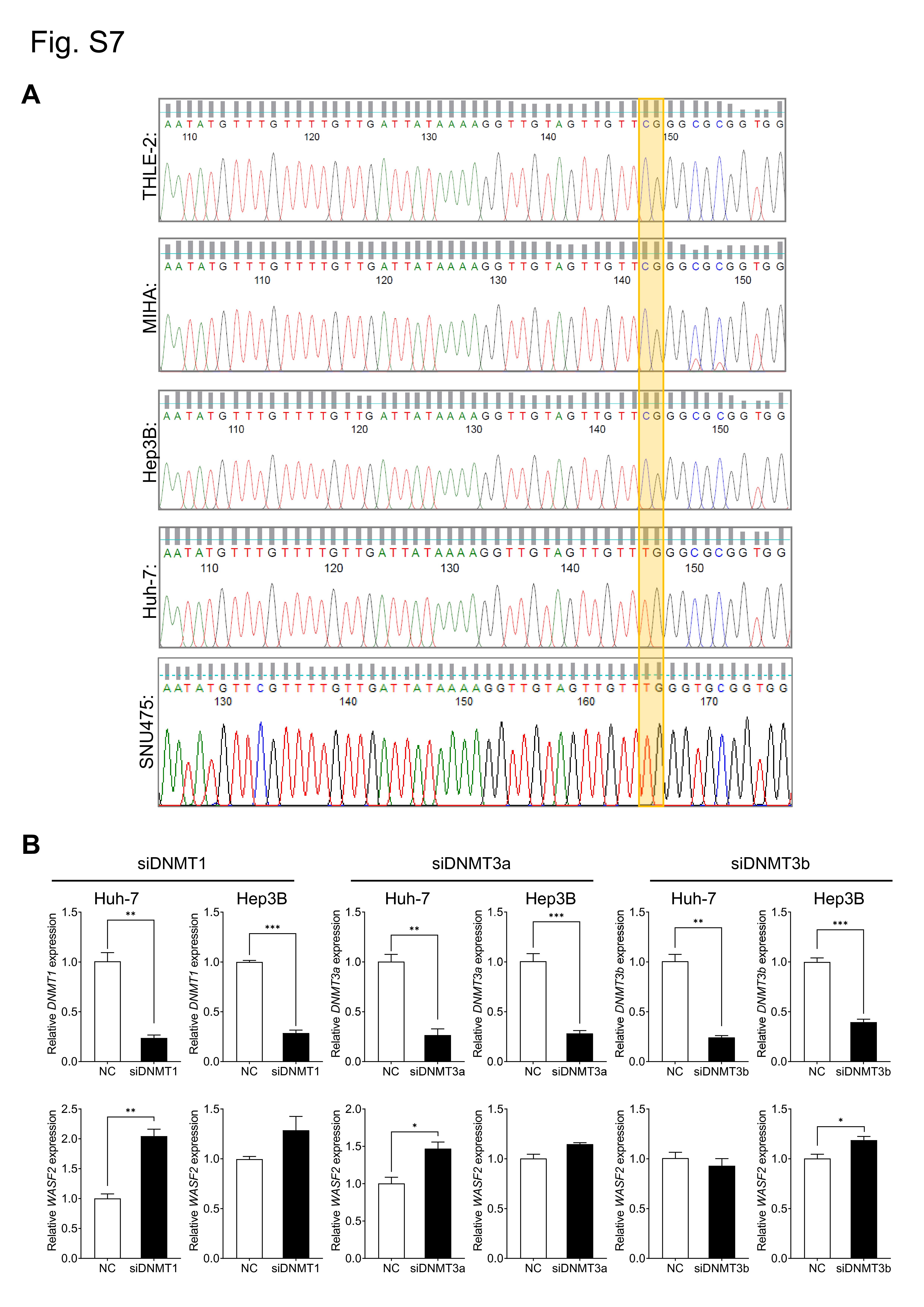


**Fig. S9 A** Direct sequencing of TA-cloned BSP products was used to determine the methylation status of each CpG island in immortalized hepatocyte cell lines (THLE-2 and MIHA) and HCC cell lines (Hep3B, Huh-7, and SNU475). **B** The efficiency of siRNAs on the silencing of DNMT1, DNMT3a, and DNMT3b in Huh-7 and Hep3B cells assessed using RT-qPCR for 48 h (top) and after transfection with siDNMT1 or siDNMT3a or siDNMT3b for 48 h, *WASF2* mRNA levels in HCC cells were evaluated through RT-qPCR (bottom).
